# Intrinsic and Extrinsic Determinants of Stability in Spiro‐OMeTAD‐Based Hole‐Transporting Layers in Perovskite Solar Cells: Mechanistic Insights and Strategic Perspectives

**DOI:** 10.1002/adma.202513270

**Published:** 2025-09-06

**Authors:** Yun Seop Shin, Jaehwi Lee, Min Jung Sung, Il Jeon, Nam Joong Jeon, Dong Suk Kim

**Affiliations:** ^1^ Graduate School of Carbon Neutrality Ulsan National Institute of Science and Technology (UNIST) UNIST‐gil 50 Ulsan 44919 Republic of Korea; ^2^ School of Energy and Chemical Engineering Ulsan National Institute of Science and Technology (UNIST) UNIST‐gil 50 Ulsan 44919 Republic of Korea; ^3^ Photoenergy Research Center Korea Research Institute of Chemical Technology (KRICT) Daejeon 34114 Republic of Korea; ^4^ Department of Nano Engineering and Department of Nano Science and Technology SKKU Advanced Institute of Nanotechnology (SAINT) Sungkyunkwan University (SKKU) Suwon 16419 Republic of Korea

**Keywords:** dopant engineering, hole‐transporting materials, long‐term stability, molecular design, perovskite solar cells, Spiro‐OMeTAD

## Abstract

Spiro‐OMeTAD has remained the benchmark hole‐transporting material (HTM) in state‐of‐the‐art perovskite solar cells, owing to its favorable energy level alignment and excellent interfacial compatibility. However, its practical implementation is critically hindered by the intrinsic instabilities introduced by conventional dopants such as lithium bis(trifluoromethanesulfonyl)imide (LiTFSI) and 4‐*tert*‐butylpyridine (*t*BP). While these dopants enhance electrical conductivity, they concurrently initiate multiple degradation pathways—including ionic migration, radical deactivation, and moisture/thermal‐induced morphological failure—thereby compromising device longevity and reproducibility. This review presents a comprehensive and mechanistic perspective on dopant‐induced instabilities in spiro‐OMeTAD‐based hole‐transporting layers, systematically unraveling the physicochemical origins of performance loss under operational stress. Recent advances in dopant design, additive engineering, and post‐oxidation‐independence doping strategies that aim to circumvent the trade‐offs inherent to traditional systems are further highlighted. Emphasis is placed on the interdependence among dopant formulation, charge transport kinetics, and environmental resilience. By integrating insights from advanced characterization and molecular‐level design, rational guidelines toward the development of next‐generation dopant systems and HTM architectures that reconcile high efficiency with long‐term operational stability are proposed. This review offers a forward‐looking framework to steer the evolution of robust and commercially viable perovskite photovoltaics.

## Graphical abstract

This review provides a comprehensive and multidimensional examination of the intrinsic and extrinsic parameters that dictate the physicochemical stability of spiro‐OMeTAD‐based hole‐transporting layers. It further underscores recent breakthroughs in dopant molecular design, additive modulation, and strategies enabling post‐oxidation‐independence doping, all of which seek to overcome the fundamental trade‐offs associated with conventional doping systems.

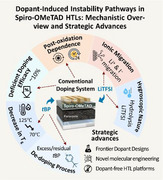

## 1 Introduction

Global energy demand continues to rise amid growing concerns over fossil fuel depletion and climate change, underscoring the urgent imperative for sustainable, low‐carbon energy solutions. Among the spectrum of renewable technologies, solar energy has assumed particular prominence, with metal halide perovskites (MHPs)^[^ ^1^, ^2^, ^3^ ^]^ emerging as a transformative class of semiconductors owing to their exceptional optoelectrical properties, including high absorption coefficients, long carrier diffusion lengths, and tunable bandgaps. These intrinsic characteristics position them as front‐running candidates for next‐generation photovoltaic technologies, particularly in the pursuit of maximizing power conversion efficiencies (PCEs) while preserving cost‐effective and scalable fabrication methodologies.

The pioneering research by Miyasaka et al. in 2009^[^ ^4^ ^]^ marked the inception of perovskite‐based photovoltaics, wherein methylammonium lead triiodide (MAPbI_3_) was utilized as a sensitizer in dye‐sensitized solar cells (DSSCs), achieving a modest PCE of 3.8%. However, the intrinsic solubility of MAPbI_3_ in the liquid electrolyte posed a severe challenge, leading to rapid decomposition and poor long‐term stability. To overcome these limitations, the field has progressed from liquid‐phase configurations to solid‐state architecture, enabling more effective control over device stability. In 2012, Park et al. initiated the development of solid‐state perovskite solar cells (PSCs) by replacing the liquid electrolyte with an organic hole‐transporting layer (HTL), specifically 2,2′,7,7′‐tetrakis[*N*,*N*‐di(4‐methoxyphenyl)amino]‐9,9‐spirobifluorene (spiro‐OMeTAD).^[^ ^5^ ^]^ This transition represented a pivotal advancement in device engineering, facilitating efficient hole extraction while simultaneously enhancing structural stability. Thereafter, the PCE rapidly escalated to 9.7%, sparking a surge in research on perovskite solar cells and heralding the onset of comprehensive investigations into their vast potential applications.^[^ ^6^, ^7^, ^8^ ^]^

Over the past decade, persistent advancements and rigorous research endeavors^[^ ^9^, ^10^, ^11^ ^]^ including charge‐transporting layer engineering, interfacial modification, and additive engineering, have culminated in the attainment of a certified PCE exceeding 26% in n‐i‐p structured PSCs. Notably, efficiency has steadily improved, driven by significant advancements in material optimization and device architecture, with the state‐of‐the‐art architecture typically comprising fluorine‐doped tin oxide (FTO)/electron‐transporting layer (ETL)/FAPbI_3_‐based perovskite/spiro‐OMeTAD/Au. A defining feature of this progress is the widespread adoption of spiro‐OMeTAD as the benchmark HTL in high‐efficiency PSCs. Its use is typically accompanied by a well‐established dopant system, composed of p‐type dopants such as 4‐*tert*‐butylpyridine (*t*BP) and lithium bis(trifluoromethanesulfonyl)imide (LiTFSI) to surmount its inherently deficient electrical characteristics,^[^ ^12^, ^13^, ^14^ ^]^ namely, its low hole mobility and poor electrical conductivity. The use of LiTFSI as a p‐type dopant in spiro‐OMeTAD HTL was first introduced by Grätzel et al. in 2006 in the context of solid‐state DSSCs (ssDSSCs),^[^ ^15^ ^]^ while the optimal 6:1 molar ratio of *t*BP to LiTFSI in spiro‐OMeTAD was established in 2011.^[^ ^16^ ^]^ Since then, this dopant formulation has been pervasively adopted in state‐of‐the‐art PSCs, thereby solidifying spiro‐OMeTAD's position as the HTL of choice for achieving high efficiency. Despite record‐setting performance metrics, challenges related to long‐term operational stability under real‐world conditions remain unresolved. Moisture‐, heat‐, and light‐induced degradation continue to pose significant obstacles to commercialization.

Spiro‐OMeTAD possesses an optical bandgap of ≈2.7 eV, a highest occupied molecular orbital (HOMO) level of ≈−5.2 eV, and a lowest unoccupied molecular orbital (LUMO) level of ≈−2.5 eV, ensuring an energetically favorable alignment with the valence band (VB) of the perovskite absorber. This optimal HOMO–VB coupling enables efficient hole extraction from the perovskite while concurrently impeding electron backflow. Furthermore, as spiro‐OMeTAD is not entirely amorphous, its semicrystalline nature and p‐type semiconducting characteristics impart a glass transition temperature (*T*_g_) of 126 °C, a melting temperature (*T*_m_) of 248 °C, and a decomposition temperature (*T*_d_) of 417 °C, thereby endowing spiro‐OMeTAD with enhanced structural integrity. These outstanding characteristics have rendered spiro‐OMeTAD an indispensable hole‐transporting material (HTM) in PSCs research.^[^ ^17^, ^18^, ^19^ ^]^

Nonetheless, the inherently nonplanar conformation of spiro‐OMeTAD, dictated by its orthogonally oriented spirobifluorene core and propeller‐like triphenylamine moieties, impedes the formation of robust *π–π* stacking. This structural limitation fundamentally restricts charge carrier delocalization, resulting in intrinsically low hole mobility (≈10^−5^ cm^2^ V^−1^ s^−1^) and poor electrical conductivity (10^−8^ S cm^−1^), necessitating strategic implementation of a p‐doping regimen through the incorporation of *t*BP and LiTFSI as dopants. The introduction of p‐doping engenders a marked enhancement in electrical conductivity (10^−4^–10^−3^ S cm^−1^), while simultaneously mitigating the band offset between the HOMO of spiro‐OMeTAD and the VB of the perovskite. However, this improvement comes at the expense of significant stability trade‐offs, which have emerged as one of the primary roadblocks to further technological advancement.

The instability issues associated with spiro‐OMeTAD‐based n‐i‐p structured PSCs have been predominantly attributed to the detrimental effects of the incorporated *t*BP and LiTFSI dopants, which exacerbate degradation pathways under environmental stressors. The incorporation of *t*BP constitutes a pivotal element within the spiro‐OMeTAD HTL system, serving as a morphological controller by enhancing the solubility of the LiTFSI p‐type dopant. However, this integration precipitates several deleterious outcomes: 1) a substantial decrease in the *T*_g_ of spiro‐OMeTAD from 125 °C to below 70 °C,^[^ ^20^, ^21^ ^]^ 2) de‐doping of spiro‐OMeTAD radicals,^[^ ^22^, ^23^ ^]^ and 3) severe degradation of the underlying perovskite layer, exacerbated by the polar characteristics of the dopant.^[^ ^24^, ^25^ ^]^ In the case of the LiTFSI p‐type dopant, 4) intrinsic hygroscopic nature poses a significant impediment to achieving long‐term humidity stability.^[^ ^26^, ^27^ ^]^ Moreover, 5) these ionic species remain non‐covalently associated with spiro‐OMeTAD, facilitating uncontrolled ion migration within the device under external perturbations such as light exposure and thermal stress.^[^ ^28^, ^29^ ^]^ Notably, the pronounced migration of Li^+^ ions, exacerbated by such external stimuli, not only compromises the structural integrity of perovskite crystals but also deteriorates the electrical properties of the ETL, either by inducing undesirable doping effects or by serving as interstitial defects that perturb charge transport dynamics. Lastly, 6) the efficacy of this dopant formulation is highly susceptible to ambient conditions, necessitating a prolonged post‐oxidation process of ≈24 h,^[^ ^30^, ^31^ ^]^ which in turn imposes a substantial bottleneck to its commercial viability.

Nevertheless, despite its intrinsic drawbacks, spiro‐OMeTAD remains the prevailing HTL of choice among researchers striving to maximize the efficiency of PSCs. This continued reliance is largely attributable to its well‐documented charge‐extraction proficiency, energetically favorable HOMO alignment with perovskite absorbers, and its pivotal role in enabling state‐of‐the‐art PCEs. However, the inherent trade‐offs associated with its structural instability, dopant‐induced degradation mechanisms, and protracted oxidation prerequisites underscore the imperative for next‐generation HTL alternatives or sophisticated doping paradigms to mitigate these limitations while preserving device performance.

Organic HTMs constitute an essential component of PSCs,^[^ ^32^, ^33^, ^34^ ^]^ with spiro‐OMeTAD serving as a benchmark material and a paradigmatic representative that has remained the focus of sustained research endeavors aimed at further advancing its performance. In this review, we transcend a mere survey of prevailing HTM research trends by systematically elucidating recent advancements in the spiro‐OMeTAD HTL system from both molecular and compositional perspectives—encompassing intrinsic spiro‐OMeTAD material, *t*BP as a volatility‐driven morphological modulator, LiTFSI as a p‐type dopant facilitating oxidative doping, and a diverse array of functional additives engineered to augment charge transport, morphological stability, and overall device performance. Furthermore, we meticulously dissect the intricate degradation mechanisms initiated by dopants, delve into the strategic integration of spiro‐OMeTAD derivatives in conjunction with various dopants and additives, and critically assess strategies aimed at mitigating the intrinsic instability constraints in spiro‐OMeTAD‐based n‐i‐p structured PSCs.

By leveraging a comprehensive classification framework, we delineate the fundamental pathways contributing to device degradation and offer an in‐depth discourse on potential avenues geared toward fortifying long‐term operational stability. In conclusion, we provide a holistic perspective on the interplay among dopant chemistry, material design, and device architecture, emphasizing their collective impact on charge transport kinetics and environmental resilience. By synthesizing insights from state‐of‐the‐art experimental investigations and theoretical paradigms, we propose rational design principles for next‐generation spiro‐OMeTAD‐based HTLs with reinforced stability and optimized optoelectronic performance. Ultimately, this review aspires to provide a comprehensive conceptual foundation for guiding future research directions, with the overarching goal of fostering scalable, durable, and high‐efficiency perovskite photovoltaics.

## 2 Conventional Spiro‐OMeTAD HTL Doping System

### 2.1 Conventional Dopants/Additives in Spiro‐OMeTAD

**Figure 1:**
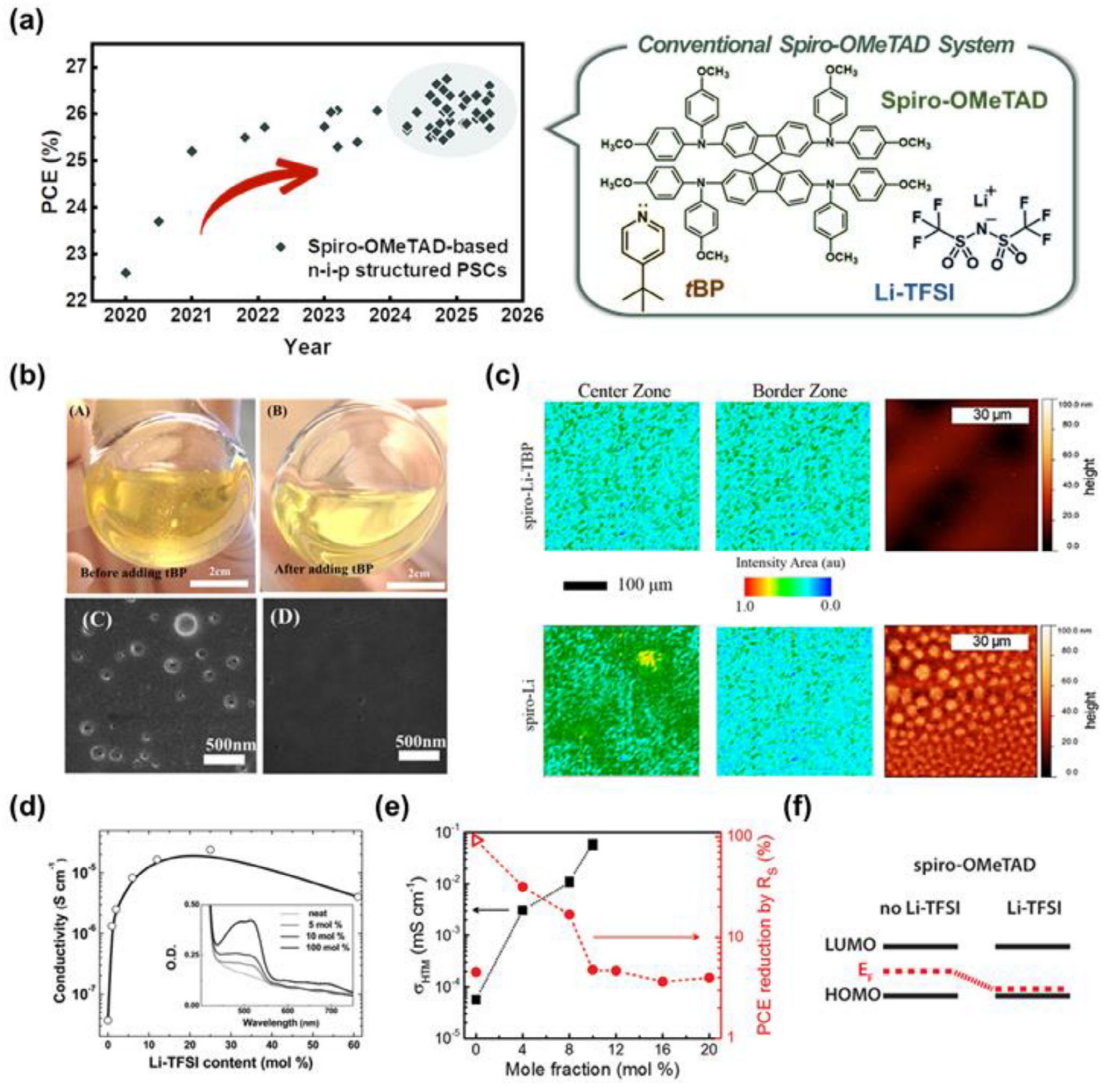
Representative dopants and additives conventionally employed in spiro‐OMeTAD‐based systems. a) Reported PCEs of PSCs utilizing spiro‐OMeTAD as the hole‐transporting layer (Table 1), along with the molecular structures of spiro‐OMeTAD and representative dopants/additives used in conventional doping formulations. b) Photographs of spiro‐OMeTAD solutions before and after the addition of *t*BP (top), and top‐view scanning electron microscopy (SEM) images of spiro‐OMeTAD films without and with *t*BP (bottom). Reproduced with permission.^[^ ^72^ ^]^ Copyright 2016, American Chemical Society. c) 2D FTIR microscopy images showing the vibrational signal at 1159 cm^−1^ corresponding to the LiTFSI salt, along with atomic force microscopy (AFM) images of spiro‐*t*BP‐Li and spiro‐Li films. Reproduced with permission.^[^ ^73^ ^]^ Copyright 2016, American Chemical Society. d) Electrical conductivity of spiro‐OMeTAD as a function of LiTFSI content, with inset showing corresponding UV–vis absorption spectra. Reproduced with permission.^[^ ^74^ ^]^ Copyright 2013, Royal Society of Chemistry. e) Variations in the electrical conductivity of spiro‐OMeTAD and corresponding PCE losses corrected for voltage drops due to total series resistance. Reproduced with permission.^[^ ^75^ ^]^ Copyright 2019, Wiley‐VCH GmbH. f) Schematic energy level diagrams of spiro‐OMeTAD with and without the incorporation of LiTFSI. Reproduced with permission.^[^ ^76^ ^]^ Copyright 2012, American Chemical Society.

Since the introduction of spiro‐OMeTAD as the HTL in 2012, replacing the liquid electrolyte, it has been proven to be pivotal in the evolution from DSSCs to solid‐state PSCs.^[^ ^7^, ^77^ ^]^ Thus, to date, the *π*‐conjugated spiro‐OMeTAD material has become an extensively adopted cornerstone in the realm of photovoltaic technologies (**Figure** **1**a and **Table** **1**), gaining particular prominence in PSCs due to its favorable HOMO alignment with the VB of perovskite, its exceptional electrical conductivity upon p‐doping, and its seamless integration with perovskite layers, compared to other HTL alternatives such as poly(triaryl amine) (PTAA) and poly(3‐hexylthiophene‐2,5‐diyl) (P3HT). Although the inherently twisted backbone of triarylamine imparts deficient electrical properties, limiting charge mobility and electrical conductivity, the efficient p‐doping process facilitated by the incorporation of *t*BP and LiTFSI dopants enables spiro‐OMeTAD to exhibit these superior characteristics. The substantial incorporation of dopants and additives, ≈56 mol% of LiTFSI and 330 mol% of *t*BP, assumes a paramount role as integral constituents within the spiro‐OMeTAD HTL, exerting a profound impact on the chemical milieu within spiro‐OMeTAD, thereby influencing its intrinsic properties (Table 1).

**Table 1:** Recent reported PCEs of PSCs utilizing spiro‐OMeTAD as the hole‐transporting layer.

Published year	Device structure	Additives in HTL	Photovoltaic parameters				Stability	Refs.
			*V*_OC_ [V]	*J*_SC_ [mA cm^−2^]	FF [%]	PCE [%]		
2022.01	FTO/paa‐QD‐SnO_2_@c‐TiO_2_/ PVK/Spiro‐OMeTAD/Au	*t*BP, LiTFSI, FK209	1.176	26.09	83.84	25.72 (25.4)^a)^	MPP tracking (LED lamp with AM 1.5G, *T*_90_ ∼350 h)	[35]
2023.01	FTO/KFSO/KFPV/ Spiro‐OMeTAD/Au	*t*BP, LiTFSI, FK209	1.17	26.12	85.22	26.04	Thermal stability (85 °C, *T*_80_ ∼500 h)	[36]
2023.02	FTO/c‐TiO_2_/3AP‐PVK/ Spiro‐OMeTAD/Au	*t*BP, LiTFSI	1.181	26.04	82.21	25.3	MPP tracking (LED lamp with AM 1.5G, *T*_90_ ∼350 h)	[37]
2023.02	FTO/SnO_2_/PACl‐PVK/ Spiro‐OMeTAD/Au	*t*BP, LiTFSI, FK209	1.178	25.69	86.15	26.08 (25.73)	MPP tracking (AM 1.5G, *T*_95_ ∼510 h)	[38]
2023.06	FTO/SnO_2_/PAD‐PVK/ Spiro‐OMeTAD/Au	*t*BP, LiTFSI, FK209	1.16	26.3	83.0	25.4 (25.0)	MPP tracking (AM 1.5G, *T*_95_ ∼1000 h) (AM 1.5G, 65 °C, 85% RH, *T*_82_ ∼500 h)	[39]
2023.10	FTO/SnO_2_/HFB‐PVK/ Spiro‐OMeTAD/Au	*t*BP, LiTFSI	1.191	26.39	82.94	26.07 (25.86)	MPP tracking (AM 1.5G, 50 °C, *T*_95_ ∼1258 h) Thermal stability (85 °C, *T*_91.1_ ∼1255 h)	[40]
2024.03	FTO/GLDA‐SnO_2_/βA/PVK/ Spiro‐OMeTAD/Au	*t*BP, LiTFSI	1.190	25.81	84.04	25.74	MPP tracking (AM 1.5G, 50 °C, *T*_91_ ∼1000 h)	[41]
2024.03	FTO/SnO_2_/Hydantoin‐PVK/ Spiro‐OMeTAD/Au	*t*BP, LiTFSI	1.167	26.184	83.962	25.656	MPP tracking (AM 1.5G, *T*_96.8_ ∼1600 h)	[42]
2024.05	FTO/SnO_2_/PVK/OLA/ Spiro‐OMeTAD/Au	*t*BP, LiTFSI, FK209	1.193	25.98	83.97	26.04 (25.16)	MPP tracking (AM 1.5G, *T*_83_ ∼1150 h)	[43]
2024.07	ITO/SnO_2_/HEHP‐PVK/ Spiro‐OMeTAD/Ag	*t*BP, LiTFSI, FK209	1.17	25.8	85.2	25.7 (25.5)	MPP tracking (AM 1.5G, *T*_98_ ∼1000 h)	[44]
2024.07	FTO/SnO_2_/AEP/PVK/ Spiro‐OMeTAD/Au	*t*BP, LiTFSI	1.171	26.39	85.42	26.40 (25.98)	MPP tracking (AM 1.5G, *T*_80_ ∼1400 h)	[45]
2024.07	ITO/SnO_2_/TFSAP‐PVK/ Spiro‐OMeTAD/Au	*t*BP, LiTFSI, FK209	1.21	25.69	82.0	25.5	Damp‐heat stability (85 °C, 85% RH, *T*_90_ ∼660 h) MPP tracking (AM 1.5 G, *T*_90_ ∼1290 h)	[46]
2024.08	FTO/c‐TiO_2_/m‐TiO_2_/PVK/ Spiro‐OMeTAD/Au	*t*BP, LiTFSI	1.181	26.41	81.84	25.53	MPP tracking (AM 1.5 G, *T*_96.6_ ∼1050 h)	[47]
2024.08	FTO/c‐TiO_2_/SnO_2_/DPA‐TFA‐PVK/Spiro‐OMeTAD/Au	*t*BP, LiTFSI, FK209	1.191	26.00	82.77	25.63	MPP tracking (AM 1.5 G, *T*_86_ ∼360 h)	[48]
2024.09	FTO/TiO_2_/PVK/ Spiro‐OMeTAD/Au	Eu(TFSI)_2_	1.210	25.41	82.50	25.45	Humidity stability (70% RH, *T*_80_ ∼5000 h) Thermal stability (85 °C, *T*_90_ ∼1000 h)	[49]
2024.09	FTO/Li‐TiO_x_/BAE/PVK/ Spiro‐OMeTAD/AU	*t*BP, LiTFSI	1.175	26.275	85.9	26.52 (26.31)	MPP tracking (AM 1.5 G, *T*_91.5_ ∼500 h)	[50]
2024.09	ITO/SnO_2_/Cs‐PVK/ Spiro‐OMeTAD/Au	*t*BP, LiTFSI	1.186	26.43	84.98	26.64 (25.94)	MPP tracking (AM 1.5 G, 85 °C, 60% RH *T*_95_ ∼2000 h)	[51]
2024.09	FTO/SnO_2_/PVK+2‐ME:CB/ Spiro‐OMeTAD/Au	*t*BP, LiTFSI, FK209	1.186	26.1	84.8	26.25 (25.5)	MPP tracking (AM 1.5 G, *T*_80_ ∼500 h)	[52]
2024.10	FTO/c‐TiO_2_/PVK/ Spiro‐OMeTAD/Au	*t*BP, LiTFSI	1.187	26.22	83.71	26.05	Light‐soaking stability (AM 1.5G, *T*_95.68_ ∼800 h)	[53]
2024.10	FTO/SnO_2_/PVK/ Spiro‐OMeTAD/Au	*t*BP, LiTFSI, FK209	1.191	26.62	84.35	26.75 (26.0)	MPP tracking (AM 1.5 G, 50 °C, *T*_81_ ∼450 h)	[54]
2024.10	FTO/SnO_2_/PVK/ Spiro‐OMeTAD/Au	*t*BP, LiTFSI, CYTFA	1.17	26.14	84.10	25.80	Thermal stability (55 °C, 55% RH, *T*_96_ ∼500 h)	[55]
2024.11	FTO/SnO_2_/NaFo/PVK/ Spiro‐OMeTAD/Au	*t*BP, LiTFSI, FK209	1.18	26.03	83.28	25.58	MPP tracking (AM 1.5 G, *T*_86_ ∼700 h)	[56]
2024.11	FTO/SnO_2_/METEAM‐PVK/ Spiro‐OMeTAD/Au	*t*BP, LiTFSI, FK209	1.173	25.92	85.82	26.11 (25.80)	Operational stability (N_2_, 55 °C, *T*_87.5_ ∼950 h)	[57]
2024.12	FTO/SnO_2_/TGC‐PVK/ Spiro‐OMeTAD/Au	*t*BP, LiTFSI	1.18	26.03	83.73	26.03	MPP tracking (AM 1.5 G, *T*_100_ ∼3000 h)	[58]
2024.12	FTO/FR‐TiO_2_/PVK/ Spiro‐OMeTAD/Au	*t*BP, LiTFSI	1.64	26.42	84.04	25.85	MPP tracking (AM 1.5 G, *T*_99_ ∼4656 h)	[59]
2024.12	FTO/SnO_2_/PVK/D18/ Spiro‐OMeTAD/Au	*t*BP, LiTFSI	1.185	26.54	83.92	26.39 (26.10)	MPP tracking (AM 1.5 G, 50 °C *T*_95.4_ ∼1100 h)	[60]
2025.01	NP‐FTO/ALD‐SnO_2_/SC‐SnO_2_/PVK/Spiro‐OMeTAD/Au	*t*BP, LiTFSI	1.203	25.98	84.49	26.40 (25.88)	MPP tracking (AM 1.5 G, 55 °C *T*_95_ ∼1200 h)	[61]
2025.01	FTO/SnO_2_/PVK/ Spiro‐OMeTAD/Au	*t*BP, LiTFSI, FK209	1.19	26.18	84.20	26.18 (26.00)	Thermal stability (85 °C, *T*_85_ ∼1000 h)	[22]
2025.02	FTO/SnO_2_/PC/PVK/ Spiro‐OMeTAD/Au	*t*BP, LiTFSI	1.186	25.43	85.56	25.80	MPP tracking (AM 1.5 G, *T*_95_ ∼350 h)	[62]
2025.02	ITO/SnO_2_/PVK/ Spiro‐OMeTAD/Au	*t*BP, LiTFSI, FK209	1.190	26.30	84.06	26.31 (26.09)	MPP tracking (AM 1.5 G, *T*_90_ ∼1000 h)	[63]
2025.04	ITO/SnO_2_/PVK/TPFS/ Spiro‐OMeTAD/MoO_x_/Ag	*t*BP, LiTFSI	1.185	25.72	84.84	25.90	MPP tracking (AM 1.5 G, 50 °C, *T*_95_ ∼900 h)	[64]
2025.04	FTO/SnO_2_/PVK/ Spiro‐OMeTAD/Au	*t*BP, LiTFSI	1.179	26.20	84.27	26.03	MPP tracking (AM 1.5 G, *T*_96.3_ ∼2000 h)	[65]
2025.05	FTO/SnO_2_/PVK/ Spiro‐OMeTAD/Au	*t*BP, LiTFSI, FK209	1.190	25.76	85.71	26.28 (25.82)	MPP tracking (AM 1.5 G, *T*_88_ ∼440 h)	[66]
2025.05	FTO/SnO_2_/PVK/ Spiro‐OMeTAD/Au	Spiro^2+^(TFSI^−^)_2_, PMPIm	1.189	25.89	84.10	25.90	MPP tracking (AM 1.5 G, 85 °C, *T*_91_ ∼1074 h)	[67]
2025.06	ITO/SnO_2_/PVK/ Spiro‐OMeTAD+C8A/Ag	*t*BP, LiTFSI	1.187	26.47	82.79	26.01 (25.68)	MPP tracking (AM 1.5 G, *T*_80_ ∼1015 h)	[68]
2025.06	FTO/TiO_2_/SnO_2_/DACl/PVK/ Spiro‐OMeTAD/Au	*t*BP, LiTFSI	1.17	26.0	84.4	25.7	MPP tracking (AM 1.5 G, 55 °C, *T*_93.6_ ∼500 h)	[69]
2025.06	FTO/SnO_2_/HS/PVK/ Spiro‐OMeTAD/Au	*t*BP, LiTFSI	1.179	26.30	85.82	26.61 (26.54)	MPP tracking (AM 1.5 G, *T*_94.9_ ∼1800 h) Thermal stability (85 °C, *T*_95.2_ ∼1800 h)	[70]
2025.06	FTO/WE‐SnO_2_/PVK/ Spiro‐OMeTAD/Au	*t*BP, LiTFSI, FK209	1.21	25.6	85.3	26.4 (26.1)	MPP tracking (AM 1.5 G, 45 °C, *T*_92_ ∼1200 h)	[71]

^a)^ Certified PCE

The *t*BP constitutes the predominant fraction of the spiro‐OMeTAD HTL system, serving as an indispensable component. However, the functional role of *t*BP diverges markedly between DSSCs and PSCs; in DSSCs, it acts as a recombination barrier through its adsorption onto the mesoporous TiO_2_ surface,^[^ ^78^, ^79^ ^]^ which serves as the photoanode, whereas in PSCs, it has been elucidated as a pivotal modulator governing the morphological evolution of the spiro‐OMeTAD film. The LiTFSI dopant is introduced into the spiro‐OMeTAD solution (dissolved in chlorobenzene [CB]) following its prior solubilization in the polar acetonitrile solvent. Wang et al. elucidated the limited solubility of the LiTFSI dopant in the spiro‐OMeTAD solution in the absence of the *t*BP additive,^[^ ^72^ ^]^ attributing this phenomenon to intrinsic solvent miscibility constraints (Figure 1b). Upon the addition of *t*BP, it ensured the formation of a morphologically homogenous spiro‐OMeTAD film, effectively mitigating phase separation. Moreover, Juarez‐Perez et al. performed microscopic analyses on the spatial distribution of Li^+^ ions and confirmed their homogeneous distribution throughout the spiro‐OMeTAD film^[^ ^73^ ^]^ (Figure 1c). *t*BP not only serves as a morphological controller but also plays a vital role in coordinating to the perovskite surface as a Lewis base ligand, was demonstrated by Habisreutinger et al. This coordination elicited an upward band bending, thereby facilitating enhanced hole extraction from the perovskite.^[^ ^80^ ^]^

Lithium salts have been extensively reported to enhance electrical conductivity across a wide spectrum of polymeric and small‐molecule organic semiconductors, serving as redox‐active p‐type dopants. In 2006, Grätzel et al. pioneered the introduction of LiTFSI as a p‐type dopant in solid‐state DSSCs, marking a significant milestone in the development of doped HTLs. Subsequently, Abate et al. elucidated the role of LiTFSI as an effective p‐type dopant in spiro‐OMeTAD, providing a detailed mechanistic understanding of its p‐doping process.^[^ ^74^ ^]^ Before functioning as a p‐type dopant, an oxygen (O_2_)‐mediated pre‐doping process is imperative, inducing the formation of spiro‐OMeTAD^•+^ radical cations. Thereafter, the stabilization of spiro‐OMeTAD^•+^ radical cations became paramount, necessitating the involvement of redox‐inactive TFSI^−^ anions. Owing to the stable charge delocalization within the TFSI^−^ body, it has played a pivotal role as a radical cation stabilizer.^[^ ^26^, ^81^, ^82^ ^]^ Furthermore, this stabilization has been instrumental in reducing the charge hopping barrier, thereby contributing to the enhancement of electrical conductivity (Figure 1d). The incorporated Li^+^ ions also serve a pivotal function beyond mere charge compensation, notably through Coulomb trap screening,^[^ ^74^, ^83^ ^]^ thereby profoundly modulating the electrical properties. Additionally, the pronounced Lewis acidity of Li^+^ ions, stemming from their strong electrostatic interactions, facilitates their coordination with electronegative moieties within spiro‐OMeTAD. This coordination perturbs the local electronic landscape, modulating the oxidative doping dynamics and inducing a Fermi level shift toward the HOMO level. Consequently, the introduction of LiTFSI as a p‐type dopant has been pivotal in attaining an exceptional fill factor (FF) by markedly diminishing series resistance (*R*_s_) through the pronounced enhancement of electrical conductivity of the spiro‐OMeTAD film^[^ ^75^ ^]^ (Figure 1e). In parallel, the meticulously engineered band alignment effectively alleviates the open‐circuit voltage (*V*_OC_) deficit (Figure 1f), thereby maximizing the attainable *V*_OC_.^[^ ^76^ ^]^

### 2.2 Conventional Doping Mechanism of Spiro‐OMeTAD

In the p‐type doping process of organic semiconductors, p‐type dopants, which accept electrons from the HOMO level of the organic semiconductor, are employed to generate hole carriers within the material. A representative example is the doping of spiro‐OMeTAD, a widely utilized HTL in PSCs, which has been extensively investigated for its oxidative doping behavior. Abate et al. elucidated a two‐step O_2_‐mediated p‐type doping mechanism that governs the formation of the radical cation species spiro‐OMeTAD^•+^TFSI^−^, as described by the following reactions:

(1) $$Spiro-OMeTAD+O_{2}⇄\;Spiro-OMeTAD^{\cdot +}O_{2}^{\cdot -}$$

(2) $$\begin{array}{ccc} Spiro-OMeTAD^{\cdot +}O_{2}^{\cdot -} & & +LiTFSI\;⇄\;Spiro-OMeTAD^{\cdot +}TFSI^{\cdot -}\\ & & +Li_{x}O_{y} \end{array}$$

**Figure 2:**
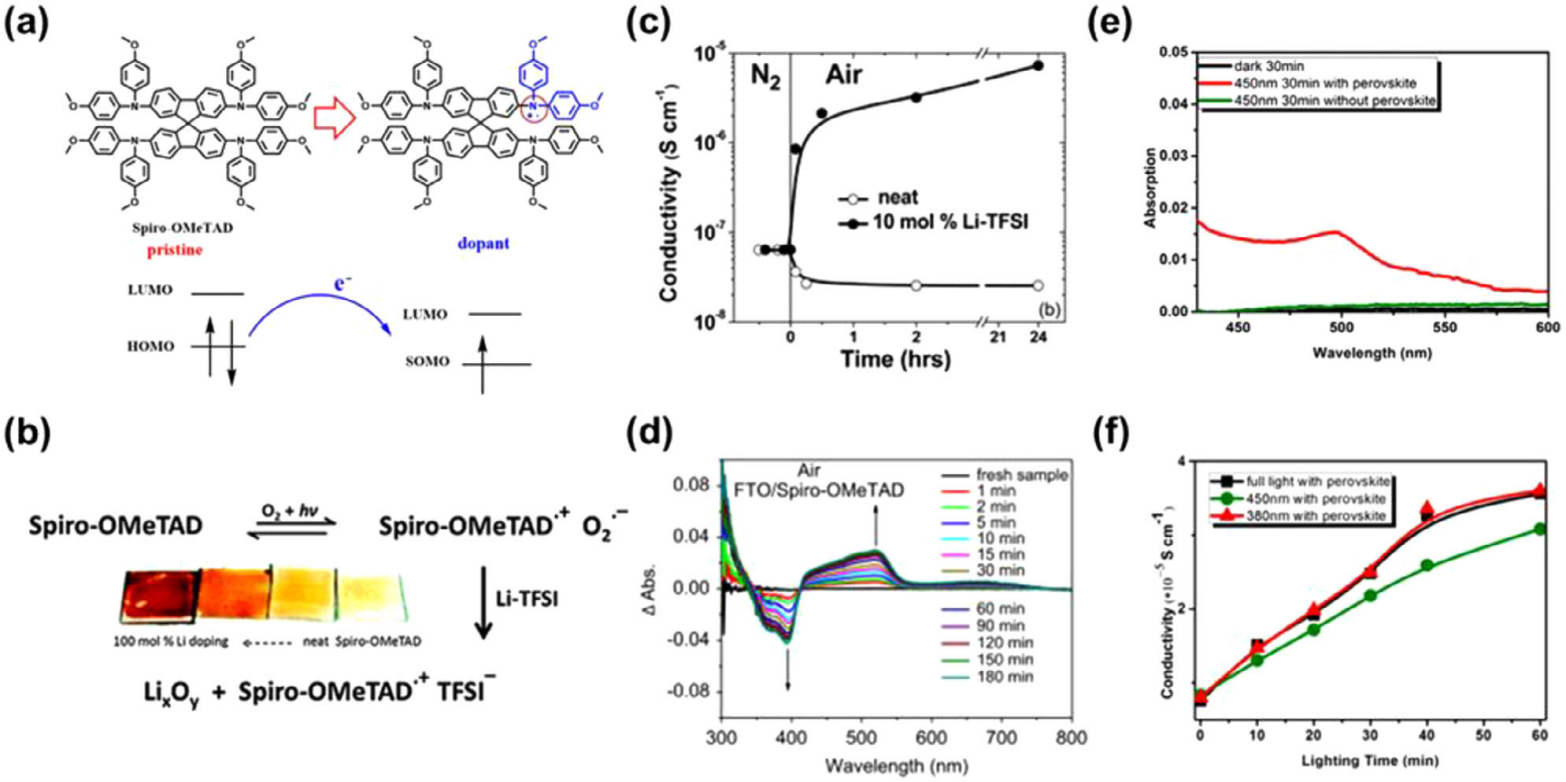
Conventional p‐type doping mechanism of spiro‐OMeTAD. a) Schematic representation of the electron transport process in spiro‐OMeTAD with molecular p‐type dopants. Reproduced with permission.^[^ ^19^ ^]^ Copyright 2020, Royal Society of Chemistry. b) Schematic illustration of the p‐type doping mechanism in spiro‐OMeTAD. c) Electrical conductivity of spiro‐OMeTAD as a function of time before (under N_2_ atmosphere) and after exposure to ambient air. Reproduced with permission.^[^ ^74^ ^]^ Copyright 2013, Royal Society of Chemistry. d) Evolution of UV–vis absorption spectra of a spiro‐OMeTAD film under 1 sun illumination (AM 1.5G, 100 mW cm^−2^) for different time intervals. Reproduced with permission.^[^ ^86^ ^]^ Copyright 2016, Elsevier. e) UV–vis absorption spectra of spiro‐OMeTAD/LiTFSI films with and without a perovskite layer after 30 min of illumination using an AM 1.5G (100 mW cm^−2^) with a 450 nm long‐pass filter (450–600 nm range). f) Electrical conductivity of spiro‐OMeTAD films measured at various illumination times and spectral ranges using a four‐point probe; hole conductivity measured in the presence of a perovskite layer. Reproduced with permission.^[^ ^88^ ^]^ Copyright 2015, American Chemical Society.

The lowest unoccupied molecular orbital (LUMO) level of spiro‐OMeTAD (−2.05 eV) is positioned energetically above the reduction potential of molecular O_2_ to superoxide (O_2_^•−^),^[^ ^84^ ^]^ which has a standard reduction potential (*E*°) of −0.35 V versus the standard hydrogen electrode (SHE). This favorable energetic alignment thermodynamically enables spontaneous electron transfer from spiro‐OMeTAD to O_2_ (**Figure** **2**a),^[^ ^85^ ^]^ facilitating the initial oxidation step in the O_2_‐mediated p‐type doping process (Equation 1). This mechanism involves an initial pre‐oxidation of spiro‐OMeTAD by molecular oxygen, generating transient radical intermediates. O_2_ serves as the primary oxidizing agent in the initial doping step, facilitating electron abstraction from spiro‐OMeTAD to form spiro‐OMeTAD^•+^O_2_^•−^ charge‐transfer complexes. Thereafter LiTFSI mediates an anion exchange reaction, wherein the O_2_^•−^ is displaced by the TFSI^−^, yielding TFSI^−^‐stabilized spiro‐OMeTAD^•+^TFSI^−^ radical species (Equation 2). This process is concomitantly accompanied by the formation of lithium oxides (Li_x_O_y_) byproducts (Figure 2b).

This conventional doping process is highly sensitive to environmental conditions, particularly the presence of oxygen,^[^ ^74^ ^]^ light irradiation,^[^ ^86^ ^]^ and humidity.^[^ ^87^ ^]^ Photoinduced excitation of electrons within spiro‐OMeTAD enhances their transfer kinetics to O_2_, thereby expediting the oxidative p‐type doping process with heightened efficacy (Figure 2c). Thus, the simultaneous presence of molecular O_2_ and incident light serves as an indispensable prerequisite for facilitating the conventional oxidative p‐type doping process of spiro‐OMeTAD (Figure 2d). In 2015, Wang et al. unveiled a spectrum‐dependent doping mechanism for spiro‐OMeTAD,^[^ ^88^ ^]^ wherein incident light within the wavelength range of 380 < λ < 450 nm possesses sufficient photon energy to exceed the bandgap of spiro‐OMeTAD. This energy is adequate to surmount the activation barrier for spiro‐OMeTAD oxidation. For incident light with wavelengths exceeding 450 nm, a perovskite‐assisted oxidation mechanism predominates (Figure 2e), wherein the formation of perovskite^•+^O_2_^•−^ complexes facilitated the oxidative doping process. Following its reaction with spiro‐OMeTAD, an analogous stabilization pathway ensued, wherein TFSI^−^ anions mediated anion exchange, ultimately generating TFSI‐stabilized spiro‐OMeTAD^•+^TFSI^−^ radicals (Figure 2f). Unlike O_2_ and light exposure, humidity serves as a deleterious factor, adversely impacting both the efficiency of the p‐type doping process and the morphological integrity of spiro‐OMeTAD. Although Hawash et al. investigated the effect of H_2_O vapor exposure on the electrical conductivity of spiro‐OMeTAD,^[^ ^87^ ^]^ the observed detrimental impact was primarily attributed to the pronounced hygroscopic nature of the LiTFSI p‐type dopant. The water uptake by the LiTFSI dopant triggers a hydration reaction, wherein the Li^+^ ions interact with H_2_O molecules, leading to the formation of LiOH and HTFSI.^[^ ^25^ ^]^ This process imparts extreme hygroscopicity and deliquescence, severely compromising the doping efficiency of spiro‐OMeTAD and ultimately deteriorating the long‐term stability of PSCs.

Nevertheless, the conventional p‐doping process is inherently sluggish, typically requiring overnight or even longer under dry air conditions due to the protracted ingress and diffusion kinetics of O_2_ into the spiro‐OMeTAD film. This diffusion‐limited mechanism renders the doping process highly susceptible to fluctuations in ambient conditions, imposing significant temporal constraints and engendering substantial irreproducibility due to environmental variability. Accordingly, alternative p‐type doping paradigms and advanced methodologies have been devised, encompassing the development of novel p‐type dopants engineered to enhance the formation of radicals, the strategic modulation of dopant interactions, and the precise regulation of environmental parameters. These approaches collectively aim to circumvent the inherent limitations of conventional doping processes, ensuring enhanced doping efficiency, reproducibility, and long‐term stability.

## 3 Limitations of Spiro‐OMeTAD Doping System

### 3.1 Deficient Doping Efficiency of Spiro‐OMeTAD

**Figure 3:**
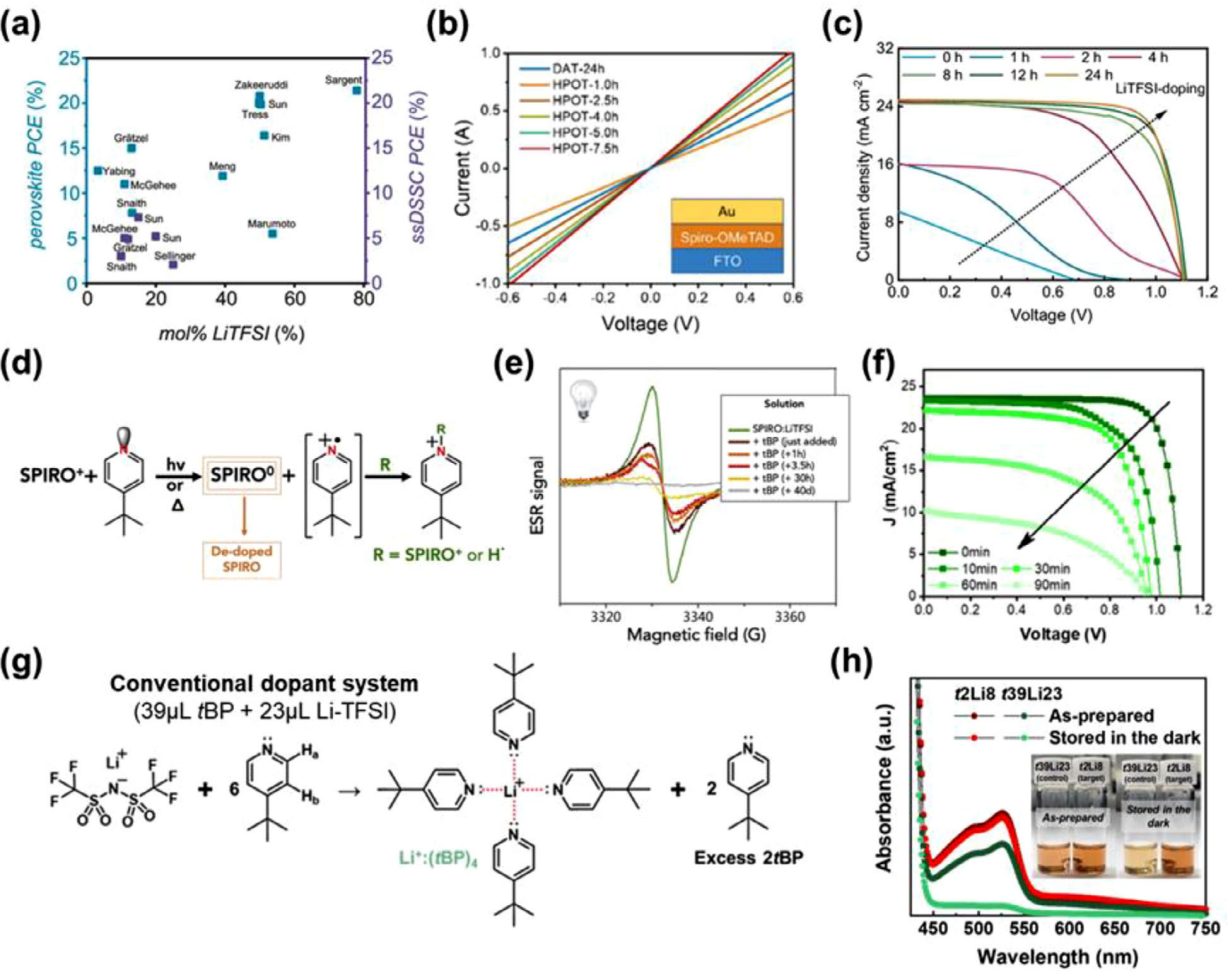
Deficient doping efficiency and de‐doping behavior of spiro‐OMeTAD. a) PCEs of PSC and ssDSSC as a function of LiTFSI concentration (mol% relative to spiro‐OMeTAD). Reproduced with permission.^[^ ^89^ ^]^ Copyright 2019, American Chemical Society. b) *I*–*V* curves of spiro‐OMeTAD films measured using hole‐only devices (FTO/spiro‐OMeTAD/Au) under N_2_ atmosphere in the dark conditions. Reproduced with permission.^[^ ^90^ ^]^ Copyright 2024, American Chemical Society. c) *J*–*V* curves of the LiTFSI‐doped PSCs as a function of oxidation time. Reproduced with permission.^[^ ^49^ ^]^ Copyright 2024, The Author(s). d) Proposed mechanism for the de‐doping of spiro‐OMeTAD in the presence of excess *t*BP. e) Electron spin resonance (ESR) analysis of the spiro‐OMeTAD molecule after the addition of *t*BP. Reproduced with permission.^[^ ^23^ ^]^ Copyright 2019, Elsevier. f) *J‐V* curves of PSCs under light‐soaking conditions using LiTFSI+*t*BP formulations. Reproduced with permission.^[^ ^91^ ^]^ Copyright 2022, American Chemical Society. g) Schematic illustration of the formation mechanism of *t*BP:Li^+^ complexes under the conventional dopant system. h) Photographs and corresponding absorption spectra of spiro‐OMeTAD solutions prepared using conventional and controlled dopant systems, measured as‐prepared and after 100 h of dark storage. Reproduced with permission.^[^ ^22^ ^]^ Copyright 2025, Elsevier.

In conventional doping systems, even when spiro‐OMeTAD is processed with substantial dopant concentrations—typically 330 mol% of *t*BP and 56 mol% of LiTFSI (**Figure** **3**a)—the actual formation of spiro‐OMeTAD^•+^TFSI^−^ radicals remains severely limited, with doping yields reported to be as low as ≈10 mol%^[^ ^89^ ^]^ This pronounced inefficiency not only underscores the limited electron transfer efficacy from spiro‐OMeTAD to oxidizing agents under standard processing conditions but also reveals the high prevalence of parasitic de‐doping pathways. Such de‐doping is primarily attributed to the intrinsic instability and high reactivity of the oxidized spiro‐OMeTAD^•+^ species, which renders them susceptible to rapid recombination or chemical quenching, especially in the presence of *t*BP.

Several factors underline the deficient doping efficiency of spiro‐OMeTAD. First, the oxidation process predominantly relies on molecular O_2_ from ambient air as a mild oxidant. However, the ingress and diffusion of O_2_ molecules into the bulk of the spiro‐OMeTAD film are severely limited by both thermodynamic and kinetic constraints^[^ ^90^ ^]^ (Figure 3b). During solution‐phase processing, the inherently low solubility of O_2_ in CB solvent—one of the most commonly employed solvents for spiro‐OMeTAD (molar fraction ≈ 7.79 × 10^−4^)—further restricts the availability of O_2_ for effective oxidative doping. As a result, the oxidation kinetics are sluggish, leading to poor and inhomogeneous radical generation within the film. Additionally, once the film is cast, the diffusion of O_2_ through the solid‐state matrix is impeded by the dense molecular packing and limited free volume within the semicrystalline spiro‐OMeTAD domains. This dual‐phase diffusion limitation—occurring both in solution and in the solid state—drastically hinders the formation rate and spatial uniformity of spiro‐OMeTAD^•+^ species. Consequently, conventional doping strategies necessitate extended post‐deposition oxidation times ranging from 12 to 24 h under ambient atmospheric conditions to reach a moderately doped state^[^ ^49^ ^]^ (Figure 3c). Even with such prolonged exposure, the doping remains suboptimal and open spatially non‐uniform.

This dependence on slow, environmentally sensitive oxidation pathways imposes substantial bottlenecks on process scalability, manufacturing throughput, and device reproducibility. The ambient oxidation process is inherently difficult to standardize due to fluctuations in humidity, oxygen partial pressure, and temperature, frequently resulting in batch‐to‐batch variation in doping profiles. Such inconsistencies compromise the electrical conductivity and charge transport properties of the HTL, ultimately leading to variability in device performance, operational stability, and long‐term reliability.

Second, another critical limitation underlying the poor doping efficiency of spiro‐OMeTAD is the de‐doping mechanism, which is predominantly facilitated by the excessive presence of *t*BP within the HTL matrix. While *t*BP plays a crucial role in solubilizing lithium salts and modulating the morphology of the spiro‐OMeTAD film, its high concentration has been increasingly recognized as a destabilizing factor for the oxidized spiro‐OMeTAD^•+^TFSI^−^ radical species (Figure 3d).

**Figure 4:**
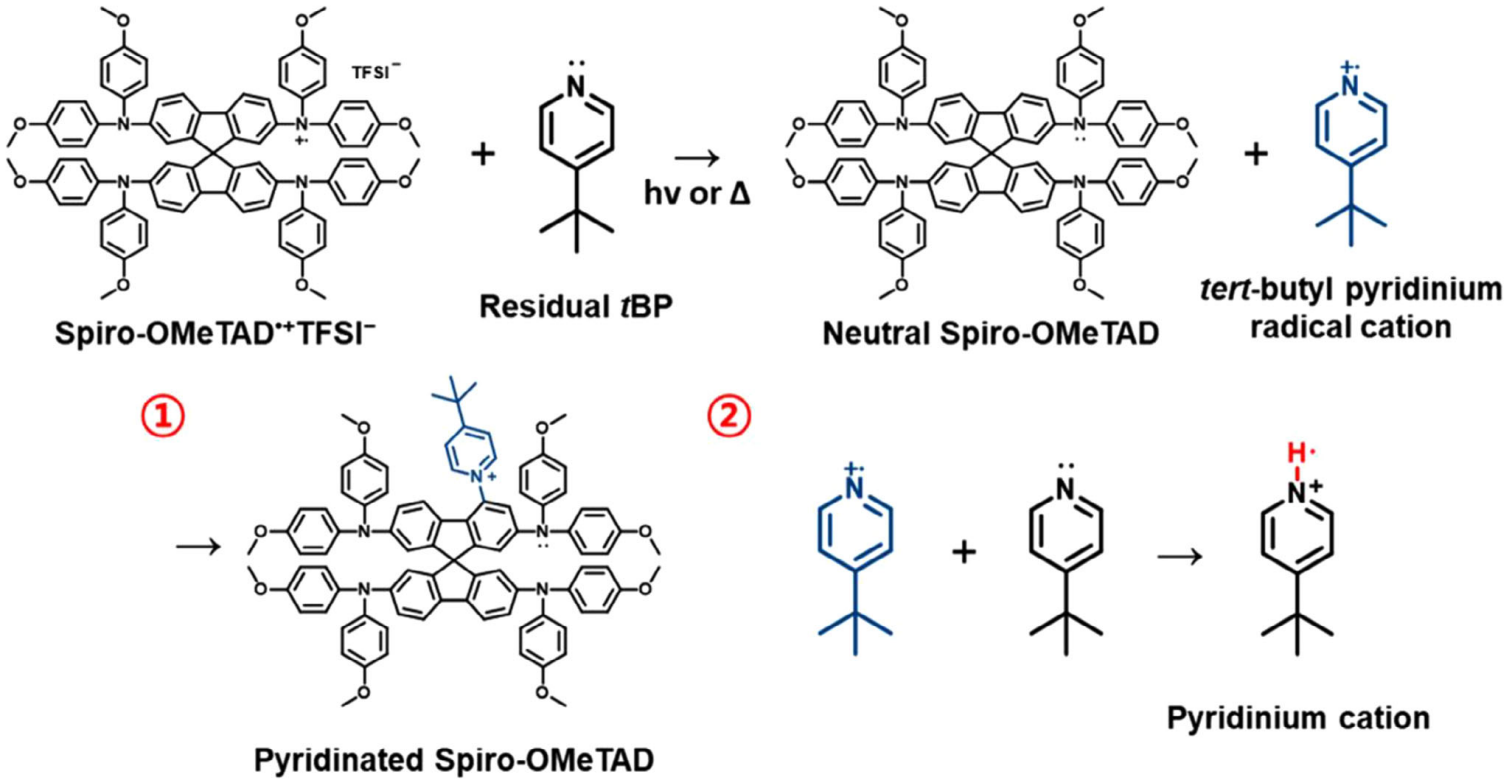
Detailed schematic illustration of the de‐doping mechanism of the spiro‐OMeTAD HTM in the presence of excess or residual *t*BP, along with the proposed byproducts formed during the de‐doping process: 1) pyridinated spiro‐OMeTAD^[^ ^93^ ^]^ and 2) pyridinium cation.^[^ ^23^ ^]^

In 2019, Lamberti et al. provided compelling spectroscopic evidence for this de‐doping mechanism through comprehensive analysis integrating electron spin resonance (ESR) and Raman spectroscopy^[^ ^23^ ^]^ (Figure 3e). Their findings demonstrated that excess *t*BP can induce a one‐electron reduction of the spiro‐OMeTAD^•+^ radical, thereby regenerating the neutral spiro‐OMeTAD molecule. This redox reaction simultaneously yields *tert*‐butyl pyridinium radical cations as reactive byproducts. These radical intermediates, due to their high instability and electrophilic nature, readily engage in secondary reactions with neighboring spiro‐OMeTAD^•+^ species (Figure 3f). This sequence culminates in the formation of pyridinated adducts,^[^ ^92^ ^]^ as later structurally characterized by Kasparavicius et al., representing irreversible degradation products that impair the electronic properties of the HTL (**Figure** **4**).

Building on these mechanistic insights, in 2025, Shin et al. revisited the conventional 6:1 molar ratio of *t*BP to LiTFSI—originally proposed in 2011 as the optimal composition—and identified it as a principal contributor to radical instability and dopant deactivation.^[^ ^22^ ^]^ According to coordination chemistry analyses conducted by Wang et al., each Li^+^ ion can coordinate with up to four *t*BP molecules, forming a relatively stable complex. Within the 6:1 formulation, however, two or more *t*BP molecules remain uncoordinated (Figure 3g), rendering them chemically available to interact with the spiro‐OMeTAD^•+^ radicals and promote reductive de‐doping. Furthermore, the weakly bound *t*BP molecules in the complex are susceptible to dissociation under thermal or ambient perturbations, such as elevated temperature, light exposure, or residual moisture. Upon release, these free *t*BP species exacerbate the de‐doping dynamics within the spiro‐OMeTAD film, accelerating the decline of radical species and compromising the long‐term stability of the HTL (Figure 3h).

### 3.2 Migration of Ionic Species

**Figure 5:**
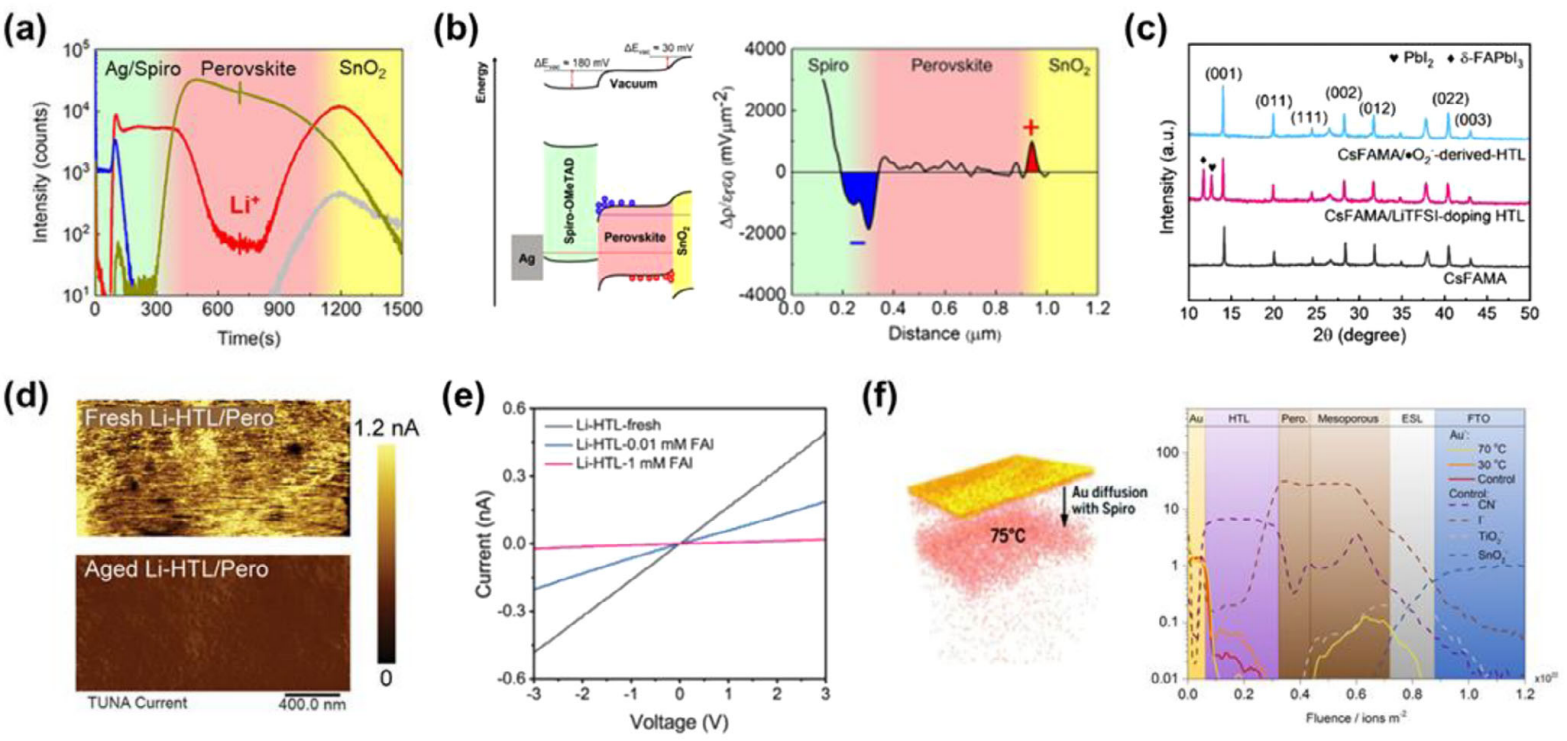
Migration behavior of ionic species in spiro‐OMeTAD‐based devices. a) Time‐of‐flight secondary ion mass spectrometry (ToF‐SIMS) depth profile of Li^+^ ions in a device subjected to aging over time. b) Accumulated charge density and schematic energy band diagrams illustrating the impact of aging on charge accumulation in devices under illumination. Reproduced with permission.^[^ ^94^ ^]^ Copyright 2023, Wiley‐VCH GmbH. c) X‐ray diffraction (XRD) patterns of HTLs deposited on perovskite films before and after aging in ambient air. Reproduced with permission.^[^ ^49^ ^]^ Copyright 2024, The Author(s). d) Surface conductivity mapping of Li‐doped HTL/perovskite before (top) and after (bottom) aging. Samples were aged under maximum power point tracking (MPPT) conditions during light soaking (AM 1.5G, 100 mW cm^−2^). e) *I–V* curves of Li‐doped HTL before and after spin‐coating with FAI solutions at concentrations of 0.01 and 1 mmol L^−1^. Reproduced with permission.^[^ ^95^ ^]^ Copyright 2022, American Association for the Advancement of Science. f) ToF‐SIMS depth profile of Au^−^ ions in devices aged under elevated temperatures. Reproduced with permission.^[^ ^96^ ^]^ Copyright 2020, Royal Society of Chemistry.

A critical and often overlooked degradation pathway in spiro‐OMeTAD‐based PSCs arises from the uncontrolled migration of Li^+^ ions. The Li^+^ ions, introduced as part of the conventional p‐type doping scheme via LiTFSI, exhibit inherently high mobility within the organic spiro‐OMeTAD matrix. These ions readily drift toward adjacent functional layers—including the perovskite absorber and the ETL—especially under external perturbations such as thermal stress, continuous illumination, or electric field bias^[^ ^29^ ^]^ (**Figure** **5**a). This ion migration results in the spatial redistribution of charges and non‐uniform interfacial ion accumulation, which significantly disrupts the electronic and chemical equilibrium across the device.

In 2017, Li et al. demonstrated that the penetration of Li^+^ ions into TiO_2_, a commonly adopted ETL, induces a dopant‐mediated electronic modulation. Specifically, Li^+^ ions alter the density of states near the conduction band of TiO_2_, enhancing charge extraction efficiency and, in some cases, improving photovoltaic performance and hysteresis behavior.^[^ ^97^, ^98^ ^]^ However, while moderate Li^+^ ion incorporation can yield beneficial effects on ETL conductivity and band structure, excessive accumulation of Li^+^ ions at the perovskite/ETL interface introduces unintended consequences. These include interfacial energy level distortion, enhanced trap‐assisted recombination, and reduced charge selectivity (Figure 5b), all of which negatively impact device performance and stability.^[^ ^99^, ^100^ ^]^ More critically, the progressive migration of Li^+^ ions through the perovskite layer has been shown to compromise the structural integrity of the crystal lattice.^[^ ^101^ ^]^ This intrusion can destabilize the perovskite framework by promoting lattice strain and disintegration, particularly in formamidinium‐based perovskites. The resultant structural stress accelerates the irreversible phase transformation of the *α*‐phase FAPbI_3_ into the photoinactive *δ*‐FAPbI_3_ polymorph (Figure 5c), undermining the optoelectronic performance of the absorber layer.

Choi et al. further revealed that such ion migration is not solely induced by operational stressors but can occur spontaneously during shelf aging, even under dark and room‐temperature conditions. Through quantitative aging studies, they established a direct correlation between Li^+^ diffusion kinetics and performance degradation, providing compelling evidence that Li^+^ migration is an intrinsic failure mechanism in the doped spiro‐OMeTAD system.^[^ ^94^ ^]^ Moreover, this process initiates a cascade of interfacial and interlayer instability phenomena. As Li^+^ ions penetrate the perovskite layer, they promote the formation of iodine vacancies and interstitial defects, which act as ion migration highways. This enhances iodide (I^−^) back‐diffusion from the perovskite into the spiro‐OMeTAD layer and, ultimately, toward the metal electrode.^[^ ^95^ ^]^ Wang et al. elucidated that the invasion of I^−^ ions triggers the chemical de‐doping of spiro‐OMeTAD^•+^TFSI^−^ species (Figure 5d), reducing hole conductivity and inducing substantial energy level misalignment. This degradation of the HTL electronic structure compromises hole extraction and contributes to voltage losses (Figure 5e).

Simultaneously, thermally activated downward migration of Au^+^ ions from the electrode interface can occur. Upon encountering I^−^ species within the spiro‐OMeTAD layer, these Au^+^ ions undergo redox reactions, forming AuI_2_^−^ complexes^[^ ^96^ ^]^ (Figure 5f). The accumulation of such reaction products not only leads to chemical decomposition of HTL but also weakens the mechanical adhesion at the Au/spiro‐OMeTAD interface, accelerating delamination and contact failure.

### 3.3 Thermal Degradation of Spiro‐OMeTAD

**Figure 6:**
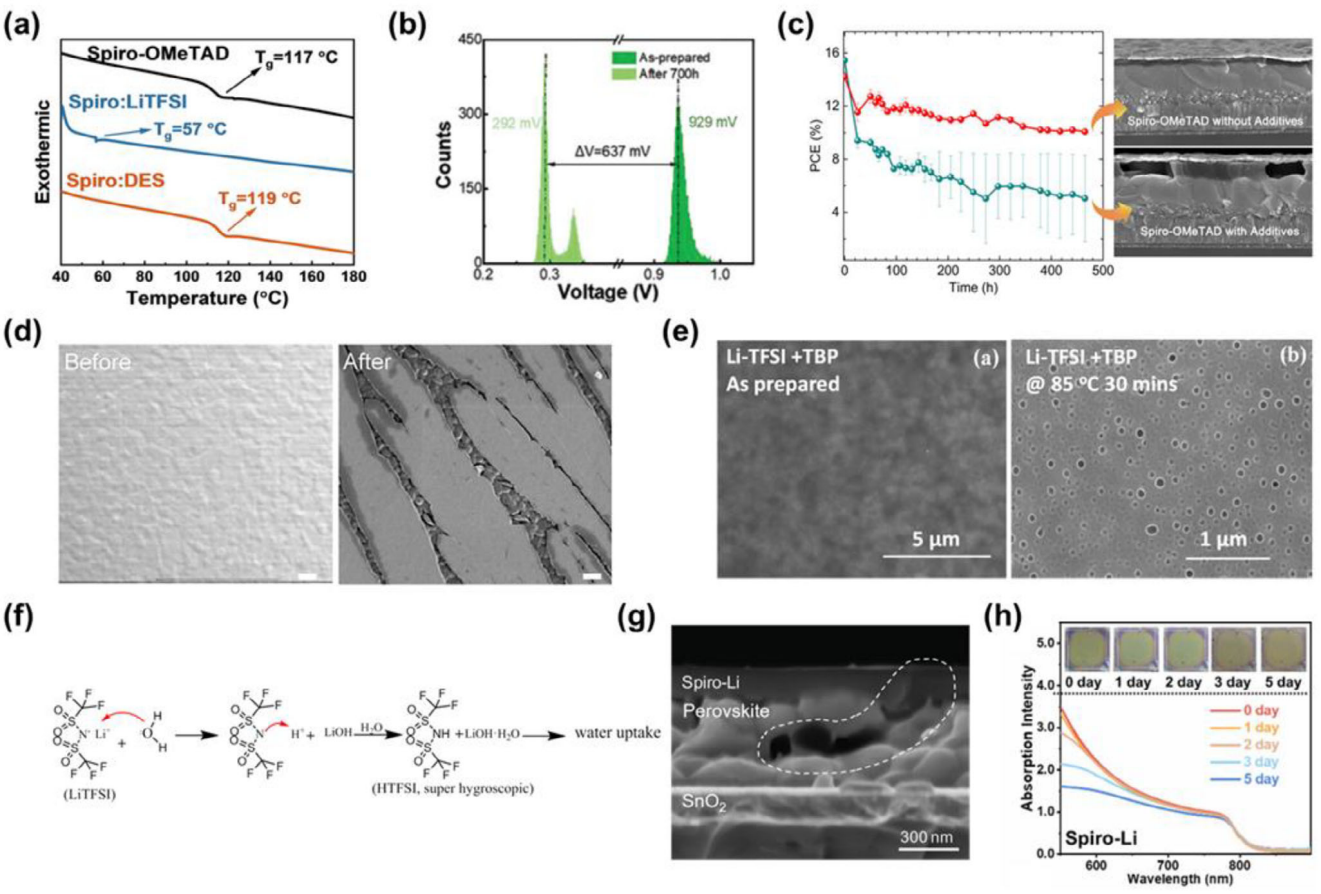
Thermal degradation and moisture‐induced instability of spiro‐OMeTAD. a) Differential scanning calorimetry (DSC) curves of pristine spiro‐OMeTAD and LiTFSI‐doped spiro‐OMeTAD. Reproduced with permission.^[^ ^102^ ^]^ Copyright 2025, Wiley‐VCH GmbH. b) Surface potential profiles of conventional spiro‐OMeTAD films before and after thermal aging. Reproduced with permission.^[^ ^113^ ^]^ Copyright 2023, Wiley‐VCH GmbH. c) Time‐dependent photovoltaic performance of spiro‐OMeTAD‐based PSCs with and without additives. Devices were unencapsulated and aged at 80 °C in ambient conditions. Reproduced with permission.^[^ ^114^ ^]^ Copyright 2020, American Chemical Society. d) Scanning electron microscopy (SEM) images of spiro‐OMeTAD films before (left) and after (right) thermal aging at 85 °C. Reproduced with permission.^[^ ^24^ ^]^ Copyright 2025, American Chemical Society. e) SEM images of freshly prepared spiro‐OMeTAD films doped with conventional LiTFSI and *t*BP (left), and after thermal stress at 85 °C for 30 min (right). Reproduced with permission.^[^ ^104^ ^]^ Copyright 2018, American Chemical Society. f) Proposed mechanism of LiTFSI hydration in the absence of *t*BP. Reproduced with permission.^[^ ^25^ ^]^ Copyright 2018, American Chemical Society. g) Cross‐sectional images of a perovskite/spiro‐OMeTAD/Ag aged for 100 h under illumination at 75 °C. Reproduced with permission.^[^ ^109^ ^]^ Copyright 2023, American Association for the Advancement of Science. h) UV–vis absorption spectra of perovskite films coated with Spiro‐Li HTLs under relative humidity (RH) ≈30%. Inset shows the morphological evolution of the corresponding films. Reproduced with permission.^[^ ^112^ ^]^ Copyright 2024, Elsevier.

In conventional n‐i‐p structured PSCs, persistent stability challenges remain, particularly under prolonged thermal stress at elevated temperatures (85 °C)—a critical threshold that simulates accelerated aging and operational reliability. While pristine spiro‐OMeTAD exhibits exceptional structural integrity, with a high *T*_g_ of 125 °C and a *T*_m_ of 248 °C, its incorporation with conventional dopants and additives fundamentally compromises its structural robustness. Among these, *t*BP acts as a potent plasticizer, profoundly lowering the *T*_g_ of spiro‐OMeTAD to below 70 °C^[^ ^102^ ^]^ (**Figure** **6**a). This reduction is strongly concentration‐dependent; higher *t*BP content enhances molecular mobility, disrupts the semicrystalline network of the spiro‐OMeTAD matrix (Figure 6b), and increases segmental chain dynamics—rendering the film highly susceptible to thermally induced morphological instability (Figure 6c).^[^ ^103^ ^]^ Under thermal stress at 85 °C, well above the compromised *T*_g_, the HTL undergoes physical deformation^[^ ^24^ ^]^ (Figure 6d), prominently characterized by surface roughening, breakdown of densification, and expansion of pinhole networks throughout the film. These pinholes serve as vulnerable diffusion channels^[^ ^104^ ^]^ (Figure 6e), enabling the escape of volatile species from the perovskite layer and, more detrimentally, facilitating the ingress of ambient moisture. The penetration of moisture initiates hydrolytic reactions with Li^+^ ions—commonly introduced via LiTFSI doping—leading to the formation of LiOH and HTFSI (Figure 6f). These byproducts not only deplete the dopant reservoir essential for sustaining the p‐doped state of spiro‐OMeTAD but also introduce unwanted ionic species that exacerbate interfacial disorder and charge imbalance.

Compounding this degradation cascade, thermal stress also disrupts the coordination complexes between Li^+^ ions and *t*BP molecules, liberating free *t*BP within the spiro‐OMeTAD matrix. These dissociated *t*BP molecules actively participate in reductive de‐doping reactions,^[^ ^93^, ^105^ ^]^ converting spiro‐OMeTAD^•+^ radicals back to their neutral form, thereby diminishing the electrical conductivity of the HTL. This degradation of charge transport capacity manifests as increased series resistance and efficiency loss under continuous operation. Moreover, the liberated *t*BP species readily diffuse toward the underlying perovskite layer, where they interact with residual PbI_2_—often present due to excess lead iodide stoichiometry. The formation of PbI_2_‐*t*BP complexes at the perovskite/HTL interface has been identified as a catalytically active degradation site.^[^ ^106^, ^107^ ^]^ These complexes facilitate the cleavage of Pb─I bonds and promote the structural decomposition of the perovskite lattice, accelerating phase segregation, ion migration, and crystal collapse. This interface‐driven decomposition pathway is particularly detrimental under dual stressors of heat and humidity, leading to rapid loss of crystallinity and optical absorption in the perovskite layer.

### 3.4 Humidity Stability of Spiro‐OMeTAD

The intrinsically poor humidity tolerance of spiro‐OMeTAD‐based PSCs—largely attributed to the highly hygroscopic nature of LiTFSI dopant—represents a formidable barrier to achieving long‐term operational stability and commercial scalability. The strong Lewis acidity of Li^+^ ions confers a high affinity toward H_2_O molecules, promoting their rapid adsorption onto the spiro‐OMeTAD matrix under ambient humidity. This interaction initiates a hydration reaction, resulting in the formation of LiOH and HTFSI—both of which destabilize the p‐doped state of the spiro‐OMeTAD matrix and catalyze further interfacial degradation.^[^ ^25^ ^]^ In addition to these primary hydration products, LiOH can further react with atmospheric CO_2_ and residual moisture to generate Li_2_CO_3_. This secondary byproduct preferentially accumulates at the spiro‐OMeTAD/perovskite interface, where it forms an electronically insulating interlayer.^[^ ^108^ ^]^ The presence of Li_2_CO_3_ has been shown to impede efficient hole extraction, weaken interfacial contact, and disrupt the energy level alignment essential for optimal charge transfer.

Exacerbating the vulnerability to moisture is the volatile nature of *t*BP, a key additive used in the conventional spiro‐OMeTAD formulations. Even under mild ambient conditions, *t*BP undergoes partial evaporation during and after film deposition, leading to the formation of microscale pinholes and voids throughout the spiro‐OMeTAD film^[^ ^109^ ^]^ (Figure 6g). These morphological defects compromise film uniformity and act as diffusion channels for moisture ingress, which in turn accelerates ionic mobility—particularly of Li^+^ and I^−^ ions—and promotes perovskite degradation processes such as hydrolysis and halide migration.^[^ ^110^, ^111^ ^]^ In addition, the continued presence of residual moisture within the HTL further accelerates the leaching of Li^+^ ions from the spiro‐OMeTAD matrix into adjacent layers such as the perovskite absorber and the metal electrode^[^ ^112^ ^]^ (Figure 6h).

Beyond the immediate effects on device performance, the pronounced sensitivity of the spiro‐OMeTAD system to humidity imposes strict environmental constraints on its practical use. Specifically, the preparation, storage, and application of spiro‐OMeTAD solutions must be carried out under inert or low‐humidity conditions to avoid premature degradation or unintentional de‐doping. These stringent requirements represent a substantial bottleneck to scalable manufacturing and roll‐to‐roll processing, as they necessitate cleanroom‐like environments for a material otherwise compatible with solution processing.

### 3.5 Uncontrollable p‐Doping of Spiro‐OMeTAD

The efficacy of p‐type doping in spiro‐OMeTAD is inherently susceptible to ambient conditions, owing to its reliance on a post‐oxidation mechanism that proceeds via molecular O_2_ and photoinduced excitation. Sanchez et al. systematically elucidated the pronounced dependence of spiro‐OMeTAD's doping kinetics on photooxidation activation,^[^ ^86^ ^]^ emphasizing its critical role in modulating photovoltaic performance metrics. Specifically, the formation of spiro‐OMeTAD^•+^ radical species is intricately governed by cumulative exposure to both molecular O_2_ and incident light, which jointly drive the oxidation of neutral spiro‐OMeTAD molecules within the solid‐state matrix. Due to this dependence, precise regulation of the oxidation atmosphere and illumination intensity during and after film deposition is imperative to attaining a uniformly and optimally doped state. When a finely tuned doping ratio is achieved, the generation of spiro‐OMeTAD^•+^ radical species markedly increases free hole carrier density within the spiro‐OMeTAD matrix, enhancing the electrical conductivity. Moreover, the improved hole density reduces localized potential barriers, facilitating more efficient hole transport and reducing recombination at the HTL/perovskite interface.

**Figure 7:**
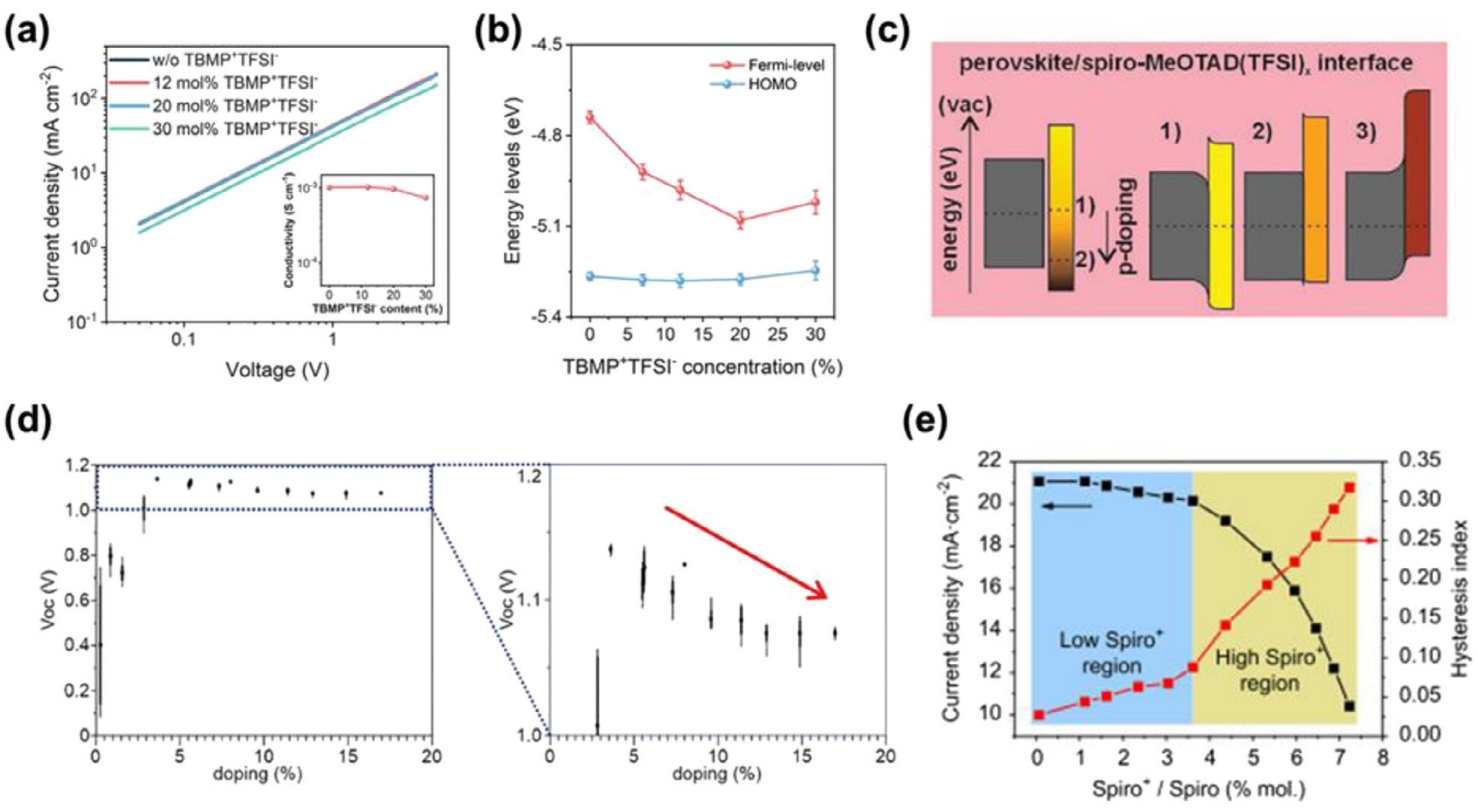
Uncontrollable p‐type doping behavior of spiro‐OMeTAD. a) *J–V* curves of hole‐only devices using spiro‐OMeTAD with varying dopant concentrations. Inset shows corresponding electrical conductivities. b) Fermi‐level and HOMO onsets of spiro‐OMeTAD films doped with different concentrations of radical cations. Reproduced with permission.^[^ ^115^ ^]^ Copyright 2022, American Association for the Advancement of Science. c) Schematic illustration of energy level alignment between the perovskite film and the spiro‐MeOTAD(TFSI)_x_ HTL. d) *V*_OC_ of devices as a function of the doping concentration of spiro‐MeOTAD(TFSI)_x_. Reproduced with permission.^[^ ^116^ ^]^ Copyright 2024, Wiley‐VCH GmbH. e) Evolution of *J*_SC_ (black squares) and hysteresis index (HI, red squares) as a function of the molar fraction of spiro‐OMeTAD^+^. The blue and beige shaded areas correspond to low and high spiro‐OMeTAD doping regimes. Reproduced with permission.^[^ ^86^ ^]^ Copyright 2016, Elsevier.

However, while moderate doping is beneficial, excessive generation of spiro‐OMeTAD^•+^ radical species—either through overexposure to light/O_2_ or overloading of LiTFSI dopant—can have deleterious consequences^[^ ^115^ ^]^ (**Figure** **7**a,b). Excess radical density leads to the emergence of trap‐like states and non‐radiative recombination centers, which accelerate electron‐hole recombination, shorten charge carrier lifetimes, and suppress FF and *V*_OC_. Additionally, excessive oxidation perturbs the band structure of the HTL, inducing band bending and energetic disorder that lead to energy level misalignment at the spiro‐OMeTAD/perovskite interface, thereby impeding efficient charge extraction^[^ ^116^ ^]^ (Figure 7c,d). Compounding this, over‐incorporation of LiTFSI dopant not only contributes to ionic instability but also results in the accumulation of Li^+^‐mediated byproducts at the perovskite/HTL interface. These interfacial species—such as LiOH, HTFSI, and Li_2_CO_3_—contribute to a significant increase in series resistance and a degradation of interfacial contact quality. The resultant barrier impairs the uninterrupted flow of hole carriers, lowering hole collection efficiency and directly compromising key photovoltaic performance metrics such as *J*_SC_ and PCE^[^ ^86^ ^]^ (Figure 7e).

Ultimately, the extreme susceptibility of the spiro‐OMeTAD doping process to environmental fluctuations—including oxygen partial pressure, light intensity, temperature, and humidity—renders the p‐doping process inherently difficult to control and poorly reproducible. This lack of control translates into batch‐to‐batch variability and poses substantial challenges for process scalability and device reliability.

## 4 Strategies to Address the Inherent Challenges

The persistent challenges impeding the commercialization of PSCs are principally ascribed to the intrinsic limitations of conventional doping paradigms, encompassing the fundamental characteristics of spiro‐OMeTAD as a HTM and the physicochemical drawbacks of widely employed dopants and additives. In response to these pressing limitations, concerted research efforts have been devoted to circumventing the inherent deficiencies of traditional doping strategies. These include reliance on post‐oxidation processes for doping activation, the deleterious migration of mobile ionic species originating from additive components, the corrosive interactions between dopants and adjacent functional layers, and the pronounced deterioration of device performance under environmental and operational stressors.

**Figure 8:**
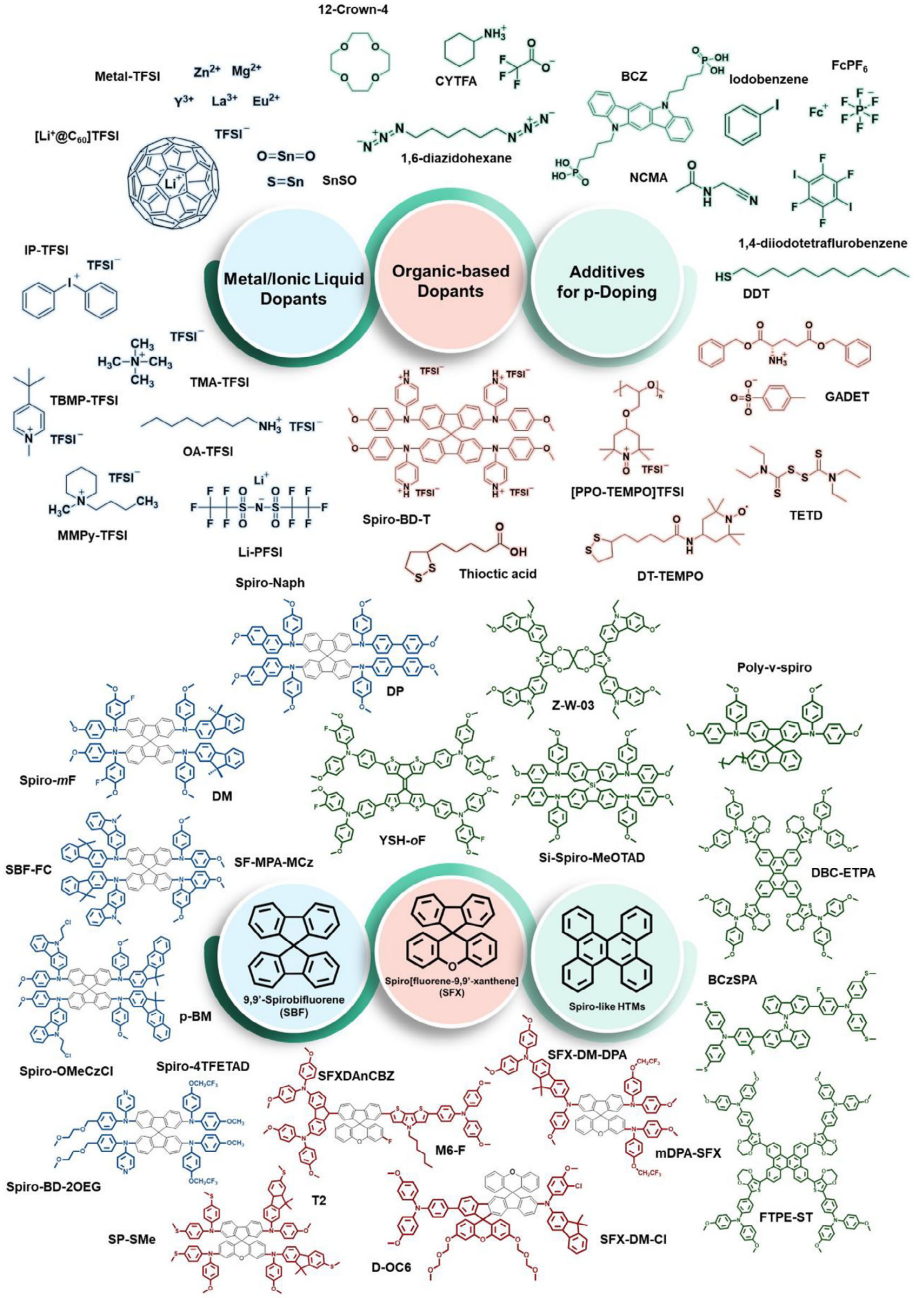
Reported molecular structures of representative alternative dopants and additives for the spiro‐OMeTAD HTL doping system, including metal/ionic liquid‐type dopants and organic molecular dopants. Reported molecular structures of representative alternative HTMs, including SBF‐based, SFX‐based, and Spiro‐like architectures.

To surmount these impediments, a multitude of innovative approaches have been pursued: the rational design of novel dopants and additives with improved chemical stability and compatibility (**Figure** **8** and **Table** **2**); the molecular tailoring of HTMs to enable efficient charge transport in the absence of extrinsic doping agents (Figure 8 and **Table** **3**); and the design of dopant‐/additive‐free hole‐transporting systems that inherently suppress ionic migration phenomena (**Table** **4**).

**Table 2:** Reported photovoltaic parameters and stability results of PSCs utilizing spiro‐OMeTAD with different additive and dopant systems.

Category	Published year	Key materials ([Dopant]/[Spiro])	Photovoltaic parameters				Stability	Refs.
			*V*_OC_ [V]	*J*_SC_ [mA cm^−2^]	FF [%]	PCE [%]		
Metal‐based	2014.09	Ag(TFSI) (0.1 mol/mol)	0.92	20.95	62.0	12.0	Long‐term stability (25 °C, 25% RH, *T*_85_ ∼2880 h)	[141]
	2019.09	[Li^+^@C_60_]TFSI (≈0.41 mol/mol)	1.09	23.1	67.0	17.2	Operational stability (AM 1.5G, 60 °C, 70% RH, *T*_100_ ∼1100 h)	[142]
	2020.03	Na(TFSI) (0.61 mol/mol)	1.12	24.5	80.5	22.4	MPP tracking (AM 1.5G, 45 °C, *T*_80_ ∼500 h)	[143]
	2020.03	Mg(TFSI)_2_ (0.44 mol/mol)	1.091	22.62	76.7	18.93	Humidity stability (55‐70% RH, *T*_83_ ∼4632 h)	[144]
	2021.08	Zn(TFSI)_2_ (0.50 mol/mol)	1.15	24.40	79.0	22.0	Long‐term stability (50 ± 5% RH, *T*_85_ ∼1440 h)	[123]
	2024.05	SnSO (‐mol/mol)	1.23	25.50	78	24.5	Thermal stability (85 ± 5 °C, *T*_82.51_ ∼1050 h) Humidity stability (50 ± 10% RH, *T*_85_ ∼1050 h)	[145]
	2024.06	Y^3+^‐*t*BP, La^3+^‐*t*BP (0.31 / 0.18 mol/mol)	1.196	24.609	82.1	24.14	MPP tracking (LED lamp with AM 1.5G, *T*_99.9_ ∼1008 h)	[124]
	2024.09	Eu(TFSI)_2_ (0.40 mol/mol)	1.210	25.41	82.50	25.45	Humidity stability (70% RH, *T*_80_ ∼5000 h) Thermal stability (85 °C, *T*_90_ ∼1000 h) MPP tracking (LED lamp with AM 1.5G, *T*_90_ ∼1000 h)	[49]
	2025.05	Li(TFSI), K(TFSI), Na(TFSI), Ca(TFSI)_2_, Mg(TFSI)_2_ (total 0.37 mol/mol)	1.194	26.36	81.75	25.78	Thermal stability (65 ± 5 °C, *T*_90_ ∼2184 h)	[125]
Ionic liquid	2020.01	PVBI‐TFSI (0.50 mol/mol)	1.16	22.99	76.0	20.33	–	[146]
	2021.03	BP‐TFSI (0.50 mol/mol)	1.13	24.4	77.6	21.3	Thermal stability (85 °C, *T*_81_ ∼500 h)	[147]
	2021.05	Li‐PFSI (0.87 mol/mol)	1.119	24.83	79.7	22.14	MPP tracking (AM 1.5G, 25 °C, 40% RH, *T*_90_ ∼1000 h)	[148]
	2022.07	TBMP‐TFSI (0.20 mol/mol)	1.175	25.52	83.88	25.1	Humidity stability (70 ± 5% RH, *T*_80_ ∼1240 h) Thermal stability (70 ± 3 °C, *T*_80_ ∼796 h)	[115]
	2023.07	DMA‐TFSI (0.01 mol/mol)	1.138	24.94	81.75	23.22	Long‐term stability (25 °C, 25% RH, *T*_80_ ∼864 h)	[149]
	2023.09	OA‐TFSI (0.17 mol/mol)	1.09	25.5	79.0	22.9	Long‐term stability (30 °C, 50% RH, *T*_85_ ∼2000 h)	[150]
	2023.12	IP‐TFSI (0.06 mol/mol)	1.19	25.07	84.35	25.16	Humidity stability (30% RH, *T*_94_ ∼2072 h) Thermal stability (85 °C, *T*_90_ ∼1000 h)	[119]
	2025.01	TMA‐TFSI (0.54 mol/mol)	1.135	25.88	78.8	23.15	Thermal stability (85 °C, *T*_80_ ∼460 h)	[126]
	2025.02	MMPy‐TFSI (0.03 mol/mol)	1.16	24.68	80.7	23.10	Thermal stability (65 °C, *T*_80_ ∼1400 h) Humidity stability (50% RH, *T*_80_ ∼1100 h)	[151]
Organic‐based	2022.02	NH_2_‐MIL‐101(Fe) (0.24 mol/mol)	1.073	23.41	75.7	19.01	Long‐term stability (25 °C, 25% RH, *T*_85_ ∼3600 h)	[152]
	2023.01	PPO‐TEMPO (0.10 mol/mol)	1.18	24.8	80.2	23.5	MPP tracking (AM 1.5G, 70 ± 5 °C, *T*_95.5_ ∼3265 h)	[109]
	2023.07	Thioctic acid (0.08 mol/mol)	1.146	24.49	80.21	22.52	Humidity stability (85‐90% RH, *T*_85_ ∼1000 h)	[153]
	2024.01	Spiro‐BD‐T (0.14 mol/mol)	1.17	24.9	82.9	24.2	Humidity stability (30% RH, *T*_90_ ∼2080 h) MPP tracking (AM 1.5G, 45–50 °C, *T*_97_ ∼1000 h)	[112]
	2024.05	GADET (0.007 mol/mol)	1.192	24.87	84.55	25.06	Humidity stability (35‐45% RH, *T*_95.02_ ∼3000 h) Thermal stability (65 °C, *T*_91.21_ ∼2000 h)	[154]
	2024.06	Spiro‐OMeTAD(TFSI)_4_ (used directly as the HTL)	1.156	25.59	81.7	24.18	MPP tracking (AM 1.5G, 85 °C, *T*_100_ ∼1400 h)	[116]
	2025.03	TETD (>100 mol/mol)	1.169	24.153	81.213	22.94	MPP tracking (AM 1.5G, 25 °C, 40% RH, *T*_88.2_ ∼1000 h)	[155]
	2025.04	DT‐TEMPO (0.30 mol/mol)	1.18	26.77	81.66	25.69	Long‐term stability (25 °C, 25% RH, *T*_100_ ∼1500 h)	[156]
Additive‐based	2022.04	12‐crown‐4 (0.17 mol/mol)	1.14	24.97	81.53	23.24	Long‐term stability (25 °C, 30–50% RH, *T*_95.9_ ∼2400 h)	[129]
	2022.06	1,6‐diazidohexane (0.11 mol/mol)	1.19	24.7	77.0	22.7	Light‐soaking stability (50 mW/cm^2^, N_2_, *T*_91_ ∼900 h)	[118]
	2022.08	1,4‐DITFB (0.02 mol/mol)	1.16	24.50	80.70	23.03	Long‐term stability (25 °C, *T*_95_ ∼1000 h)	[107]
	2022.12	DDT (0.18 mol/mol)	1.198	25.9	79.2	24.6	MPP tracking (AM 1.5G, 30–35 °C, *T*_90_ ∼1000 h)	[110]
	2023.06	Liquid crystal (‐mol/mol)	1.151	25.63	82.76	24.42	Long‐term stability (25 °C, 25% RH, *T*_91_ ∼1700 h)	[157]
	2023.12	BCZ (0.02 mol/mol)	1.16	25.65	81.99	24.51	Long‐term stability (35‐45% RH, *T*_90_ ∼3000 h) Humidity stability (30 °C, 65 ± 5% RH, *T*_80.59_ ∼800 h)	[158]
	2024.03	Iodobenzene (0.05 mol/mol)	1.174	26.07	84.18	25.76	Long‐term stability (25 °C, 25% RH, *T*_91.86_ ∼2000 h)	[120]
	2024.09	FcPF_6_ (0.01 mol/mol)	1.18	25.59	82.71	25.02	MPP tracking (AM 1.5G, 65 °C, 50% RH, *T*_95_ ∼1500 h)	[130]
	2024.10	CYTFA (‐mol/mol)	1.17	26.14	84.10	25.80	Thermal stability (55 °C, 55% RH, *T*_96_ ∼500 h)	[55]
	2025.02	NCMA (‐mol/mol)	1.18	25.51	82.91	25.02	Thermal stability (85 °C, 40% RH, *T*_90_ ∼1200 h)	[102]

**Table 3:** Reported photovoltaic parameters and stability results of PSCs utilizing molecularly designed HTMs.

Category	Published year	Key materials	Photovoltaic parameters				Stability	Refs.
			*V*_OC_ [V]	*J*_SC_ [mA cm^−2^]	FF [%]	PCE [%]		
SBF‐based HTMs	2018.07	DM	1.14	24.91	81.29	23.2	Thermal stability (60 °C, 25% RH, *T*_95_ ∼500 h)	[134]
	2020.09	Spiro‐*m*F	1.164	26.35	80.90	24.82	Humidity stability (50% RH, *T*_87_ ∼500 h)	[140]
	2020.12	SC	1.15	23.47	80.62	21.76	Long‐term stability (25 °C, 30 ± 5% RH, *T*_90_ ∼720 h) Thermal stability (60 °C, N_2_, *T*_87_ ∼500 h)	[159]
	2021.12	Spiro‐4TFETAD	1.17	24.31	74.26	21.11	Humidity stability (25 °C, 60% RH, *T*_83_ ∼250 h) MPP tracking (AM 1.5G, 40 °C, *T*_82_ ∼100 h)	[160]
	2022.01	Spiro‐Naph	1.16	25.97	80.60	24.43	Thermal stability (60 °C, N_2_, *T*_78.5_ ∼400 h)	[161]
	2022.08	Spiro‐carbazole	1.18	24.56	76.0	22.01	Thermal stability (60‐80 °C, *T*_70_ ∼220 h) Humidity stability (60% RH, *T*_80_ ∼480 h)	[162]
	2022.09	Spiro‐BD‐2OEG	1.17	25.36	81.54	24.19	Humidity stability (30% RH, *T*_89_ ∼1000 h) Thermal stability (85 °C, *T*_83_ ∼602 h) MPP tracking (AM 1.5G, 45–50 °C, *T*_90_ ∼800 h)	[163]
	2023.05	DP	1.14	26.13	84.90	25.24	MPP tracking (LED lamp with AM 1.5G, 65 °C, 85% RH, *T*_87_ ∼600 h)	[164]
	2023.06	SBF‐FC	1.18	25.90	80.90	24.70	Thermal stability (85 °C, *T*_92_ ∼500 h)	[131]
	2024.01	Spiro‐BD‐T	1.17	24.9	82.9	24.2	Humidity stability (30% RH, *T*_90_ ∼2080 h) MPP tracking (LED lamp with AM 1.5G, 45–50 °C, *T*_97_ ∼1000 h)	[112]
	2024.03	SF‐MPA‐MCz	1.18	26.24	79.20	24.53	Thermal stability (85 °C, *T*_85_ ∼1000 h) MPP tracking (AM 1.5G, *T*_90_ ∼500 h)	[165]
	2024.08	Spiro‐4	1.17	24.98	80.00	23.38	Long‐term stability (30 °C, 10% RH, *T*_97_ ∼2400 h)	[166]
	2024.12	Spiro‐mCl	1.16	26.09	84	25.26	Thermal stability (65 °C, *T*_71_ ∼240 h)	[167]
	2024.12	Spiro‐OMeCzCl	1.159	26.23	80.62	24.60	Thermal stability (60‐90 °C, 50% RH, *T*_83_ ∼204 h) MPP tracking (AM 1.5G, *T*_92.55_ ∼500h)	[168]
	2025.01	p‐BM	1.184	25.77	83.56	25.49	Humidity stability (30‐40% RH, *T*_93.8_ ∼4000 h) Thermal stability (85 °C, *T*_80.4_ ∼500 h) MPP tracking (AM 1.5G, *T*_97.9_ ∼1,000h)	[169]
SFX‐based HTMs	2020.06	SFXDAnCBZ	1.09	23.10	83	20.87	Long‐term stability (30 °C, 30% RH, *T*_77_ ∼720 h)	[170]
	2022.07	M6‐F	1.154	24.45	78.56	22.17	Light‐soaking stability (AM 1.5G, 40 °C, *T*_86_ ∼816 h) Humidity stability (30% RH, *T*_88_ ∼1000 h) Thermal stability (60 °C, *T*_97_ ∼200 h)	[171]
	2022.10	SFX‐DM‐DPA	1.15	24.5	80.6	22.70	Long‐term stability (65 °C, 45% RH, *T*_80_ ∼1000 h)	[172]
	2023.11	D‐OC6	1.17	25.27	83.71	24.80	MPP tracking (AM 1.5G, 20–30% RH, *T*_96_ ∼400 h)	[173]
	2024.02	SP‐SMe	1.16	24.23	77.65	21.95	Humidity stability (35 ± 5% RH, *T*_90_ ∼500 h) Thermal stability (65 °C, *T*_85_ ∼160 h)	[174]
	2024.05	SFX‐DM‐Cl	1.17	25.20	84.03	24.80	Light‐soaking stability (AM 1.5G, 25% RH, *T*_83_ ∼500 h)	[175]
	2024.06	T2	1.175	26.47	84.94	26.41	Light‐soaking stability (AM 1.5G, *T*_95_ ∼600 h) Thermal stability (60 °C, *T*_84_ ∼1500 h)	[176]
	2024.08	mDPA‐SFX	1.18	25.50	82.54	24.80	Humidity stability (40% RH, *T*_87_ ∼500 h) MPP tracking (AM 1.5G, 30 °C, *T*_80_ ∼2238 h)	[177]
Spiro‐like HTMs	2023.07	YSH‐*o*F	1.15	25.56	80.24	23.59	Thermal stability (60 °C, *T*_84_ ∼500 h) Light‐soaking stability (AM 1.5G, 40 °C, *T*_80_ ∼500 h)	[178]
	2023.09	BDT‐C8‐3O	1.163	25.51	81.31	24.11	MPP tracking (AM 1.5G, 45–55 °C, *T*_84_ ∼2000 h) Thermal stability (85 °C, *T*_79.5_ ∼2000 h)	[179]
	2023.11	Si‐Spiro‐MeOTAD	1.12	25.9	77.1	22.5	MPP tracking (AM 1.5G, *T*_92.9_ ∼120 h)	[180]
	2024.02	Poly‐v‐spiro	1.173	25.79	81.11	24.54	Humidity stability (23 ± 2 °C, 50 ± 10% RH, *T*_90_ ∼2000 h) Light‐soaking stability (AM 1.5G, *T*_95_ ∼1250 h)	[181]
	2024.02	FTPE‐OSMe	1.14	26.31	83.37	24.94	Thermal stability (60 °C, 25% RH, *T*_90_ ∼1000 h) MPP tracking (AM 1.5 G, 25 °C, *T*_90_ ∼800 h)	[182]
	2024.04	DBC‐ETPA	1.143	25.97	83.17	24.7	Thermal stability (85 °C, *T*_88_ ∼1000 h) MPP tracking (AM 1.5G, 45 °C, *T*_94_ ∼500 h)	[132]
	2024.04	BCzSPA	1.17	25.99	83.81	25.42	Thermal stability (85 °C, *T*_80.3_ ∼1000 h) MPP tracking (AM 1.5G, 45 °C, *T*_82.2_ ∼2400 h)	[183]
	2024.05	FTPE‐ST	1.136	26.24	84.6	25.21	Thermal stability (60 °C, 25% RH, *T*_80_ ∼1000 h) MPP tracking (AM 1.5G, 60 °C, *T*_83_ ∼600 h)	[135]
	2024.08	Z‐W‐03	1.18	24.72	82.50	24.02	MPP tracking (AM 1.5G, *T*_95.5_ ∼ 1500 h) Humidity stability (20‐30% RH, *T*_95.05_ ∼1440 h)	[184]

**Table 4:** Recent reported PCEs of PSCs utilizing *t*BP‐free spiro‐OMeTAD as the hole‐transporting layer.

Published year	Additives/Dopants ([Dopant]/[Spiro])	Photovoltaic parameters				Refs.
		*V*_OC_ [V]	*J*_SC_ [mA cm^−2^]	FF [%]	PCE [%]	
2017.06	TBA‐TFSI (0.54 mol/mol)	1.071	22.3	77	18.4	[104]
2017.08	Mo(tfd‐COCF_3_)_3_ (0.05 mol/mol)	1.07	22.11	76	17.8	[185]
2018.09	BMPy‐TFSI (0.08 mol/mol)	1.020	21.17	65.12	14.06	[186]
2018.09	Cu(dpm)_2_(PF_6_)_2_ (0.09 mol/mol)	1.12	22.8	75	19.3	[187]
2020.05	TPFB (0.12 mol/mol)	1.141	23.44	75.29	20.10	[188]
2020.06	PFPPY (0.15 mol/mol)	1.12	23.98	79.62	21.38	[189]
2021.09	Sb_2_S_3_ (0.02 mol/mol)	1.132	24.75	79	22.13	[190]
2022.06	POM@MOF (0.007 mol/mol)	1.11	24.1	80.0	21.5	[191]
2022.07	DIC‐PBA (0.05 mol/mol)	1.14	24.29	82.1	22.73	[192]
2022.07	TBMP‐TFSI & Spiro‐OMeTAD(TFSI)_2_ (0.20 mol/mol)	1.175	25.52	83.88	25.15	[115]
2024.01	IP‐TFSI (0.06 mol/mol)	1.19	25.07	84.35	25.16	[119]
2024.06	Spiro‐OMeTAD(TFSI)_x_ (used directly as the HTL)	1.156	25.59	81.7	24.18	[116]
2025.02	NCMA (‐ mol/mol)	1.18	25.51	82.91	25.02	[102]
2025.02	Ethylene carbonate (0.05 mol/mol)	1.180	26.031	83.18	25.56	[133]

In line with dopant engineering strategies, recent efforts have explored spiro‐OMeTAD derivatives as alternative HTMs with intrinsically enhanced optoelectronic properties and environmental resilience. These derivatives are structurally tailored through backbone modifications, side‐chain substitutions, or heteroatom integration to improve charge transport, energy‐level alignment, and morphological stability. Notably, several spiro‐OMeTAD derivatives have demonstrated superior thermal and film‐forming stability, mitigating performance degradation typically associated with dopant‐induced failures. From an application standpoint, such molecularly engineered HTMs have achieved promising performance in both small‐area and scalable PSC configurations, with PCEs exceeding 25% in some cases. Their compatibility with dopant‐free or low‐dopant formulations further enhances device reproducibility and operational longevity, positioning spiro‐OMeTAD derivatives as key components for next‐generation HTL architectures that satisfy the performance, stability, and scalability requirements of practical photovoltaic applications.

Collectively, these strategies endeavor to fortify the intrinsic durability, efficiency, and scalability of PSCs, thereby accelerating their trajectory toward practical deployment. From a practical deployment standpoint, due consideration must be given to the prohibitive synthesis cost and the intricacy of the multi‐step fabrication protocol associated with spiro‐OMeTAD. Such constraints not only exacerbate the overall manufacturing expenditure of PSCs but also hinder scalability, thereby catalyzing the impetus toward alternative HTMs that embody streamlined synthetic pathways, reduced material expenditure, and optoelectronic attributes commensurate with or exceeding those of spiro‐OMeTAD.

### 4.1 Post‐Oxidation‐Independent Doping Strategy

Within the conventional doping paradigm of spiro‐OMeTAD, attaining a sufficient doping level typically necessitates an extended post‐oxidation period of 12–24 h, thereby posing significant challenges to realizing controlled and efficient oxidation. To accelerate the doping kinetics of spiro‐OMeTAD to within a few hours while decoupling the process from environmental constraints, considerable efforts have been directed toward optimizing ambient conditions and developing advanced additive‐mediated doping strategies.

#### 4.1.1 Regulation of Ambient Parameters

**Figure 9:**
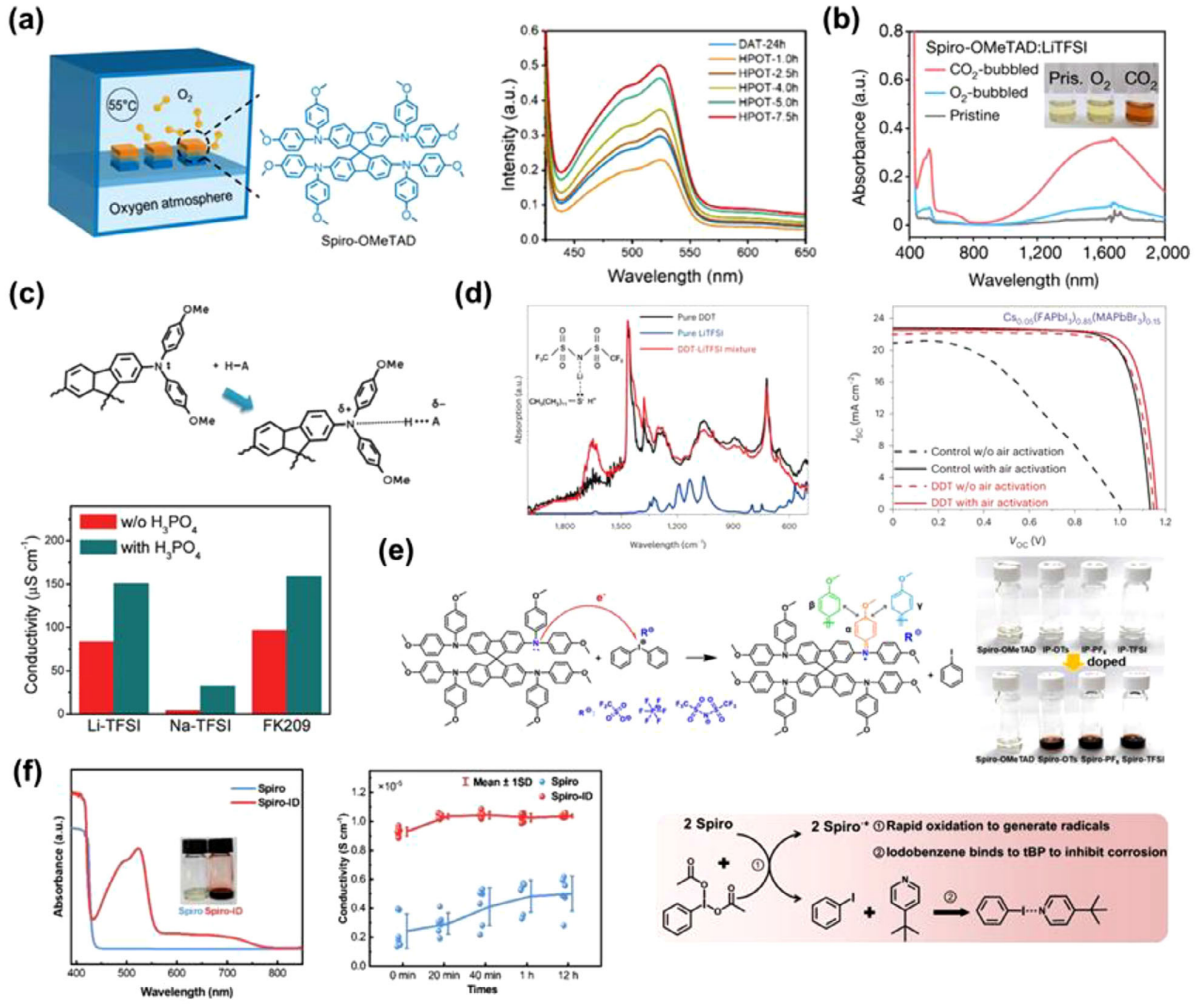
Post‐oxidation‐independent doping strategy. a) Schematic illustration of n‐i‐p PSC devices and the hot pure oxygen treatment (HPOT) process. Corresponding UV–vis absorption spectra of spiro‐OMeTAD solutions obtained by redissolving post‐treated spiro‐OMeTAD films. Reproduced with permission.^[^ ^90^ ^]^ Copyright 2024, American Chemical Society. b) UV–vis absorption spectra of pristine, O_2_‐bubbled and CO_2_‐bubbled spiro‐OMeTAD:LiTFSI solutions. Reproduced with permission.^[^ ^108^ ^]^ Copyright 2021, The Author(s). c) Schematic representation of the interaction between acidic species and spiro‐OMeTAD molecules (top); corresponding conductivities of spiro‐OMeTAD thin films with different dopant formulations (bottom). Reproduced with permission.^[^ ^117^ ^]^ Copyright 2016, Wiley‐VCH GmbH. d) FTIR spectra of a DDT‐LiTFSI mixture (red), pristine LiTFSI (blue), and DDT (black). Inset shows schematic illustration of LiTFSI coordination with DDT molecules. Also shown are *J–V* curves of PSC devices using pristine and DDT‐engineered spiro‐OMeTAD, with and without air activation. Reproduced with permission.^[^ ^110^ ^]^ Copyright 2023, The Author(s). e) Schematic diagram of the oxidation mechanism of spiro‐OMeTAD using iodonium‐based initiators. Photographs of pristine and iodonium‐doped spiro‐OMeTAD solutions stored in an N_2_‐filled glove box after 24 h. Reproduced with permission.^[^ ^119^ ^]^ Copyright 2023, Wiley‐VCH GmbH. f) UV–vis absorption spectra and corresponding conductivity evolution over oxidation time for Spiro and Spiro‐ID solutions. Schematic representation of the dual‐function cascade reaction mechanism enabled by ID. Reproduced with permission.^[^ ^120^ ^]^ Copyright 2024, Wiley‐VCH GmbH.

The inherently low solubility of O_2_ in CB solvent, coupled with its sluggish diffusion kinetics within the spiro‐OMeTAD matrix, severely limits the generation of spiro‐OMeTAD^•+^ radical species, thereby impeding the attainment of high doping efficiency. The adequate penetration of O_2_ facilitates the post‐oxidation reaction of spiro‐OMeTAD, accelerating the generation of spiro‐OMeTAD^•+^ radical species. This, in turn, enables the expeditious realization of superior electrical conductivity while concurrently mitigating the challenges associated with its doping reproducibility. In 2024, Gao et al. proposed a thermally assisted pure oxygen post‐treatment strategy to expedite the oxidation doping kinetics of spiro‐OMeTAD^[^ ^90^ ^]^ (**Figure** **9**a). This approach leverages controlled thermal activation at a moderate heating of 55 °C, which enhances the kinetic energy and diffusivity of molecular O_2_. The elevated molecular mobility facilitates deeper and more facile penetration of O_2_ molecules into the spiro‐OMeTAD matrix, thereby promoting efficient charge transfer and generation of hole polarons throughout the film. As a result, this strategy increases the doping rate and enables the attainment of high electrical conductivity surpassing 10^−3^ S cm^−1^ within just 5 h—considerably reducing the doping time compared to conventional air oxidation processes. Moreover, the improved charge transport characteristics lead to a substantial reduction in series resistance, ultimately culminating in a record‐certified fill factor exceeding 87% and a certified PCE of 25.34%.

In 2021, Kong et al. spearheaded the utilization of carbon dioxide (CO_2_) as an alternative oxidizing agent to molecular O_2_, for promoting the expedited p‐type doping of spiro‐OMeTAD in solution^[^ ^108^ ^]^ (Figure 9b). This strategy leverages the significantly higher solubility of CO_2_ in CB—approximately ten times higher than that of O_2_—allowing for a more homogeneous and sustained delivery of the oxidant throughout the spiro‐OMeTAD/LiTFSI solution. Additionally, CO_2_ possesses a favorable reduction potential, facilitating its photo‐reduction to the CO_2_^•−^ radical anion under light irradiation. This reactive intermediate serves as a potent oxidizing species capable of initiating the electron transfer required to generate spiro‐OMeTAD^•+^ radical cations. Consequently, CO_2_ bubbling under illumination leads to rapid and uniform oxidation, achieving up to a 100‐fold increase in the electrical conductivity of the resulting spiro‐OMeTAD film. Beyond enhancing doping efficiency, this CO_2_‐based strategy also offers improved reproducibility and greater environmental compatibility compared to conventional air‐based aging strategies. Moreover, the versatility of this approach extends beyond small‐molecule dopants, demonstrating promising applicability to a range of *π*‐conjugated polymeric semiconductors, thereby broadening their relevance across diverse classes of optoelectronic materials.

#### 4.1.2 Expedited Doping Strategy via Functional Additives

In 2017, Zhu et al. introduced an acid‐assisted doping strategy, in which phosphoric acid (H_3_PO_4_) served as a catalytic agent to accelerate the oxidation doping of spiro‐OMeTAD^[^ ^117^ ^]^ (Figure 9c). This approach leveraged the interaction between the acidic protons of H_3_PO_4_ and the amine moieties of spiro‐OMeTAD, where weak hydrogen bonding induced a localized polarization effect. This polarization facilitated charge delocalization and promoted a favorable energetic landscape for hole generation, especially in the presence of alkali metal salts such as LiTFSI. The resulting enhancement in hole carrier density significantly improved the electrical conductivity of the doped spiro‐OMeTAD film. Han et al., in 2022, reported a novel azide‐functionalized small molecule, 1,6‐diazidohexane (N3), as an effective dopant integrated into the conventional *t*BP/LiTFSI doping matrix.^[^ ^118^ ^]^ The azido moiety in N3 exhibited strong dipolar characteristics, engaging in permanent dipole‐dipole intermolecular interactions with both *t*BP and LiTFSI. These interactions enhanced the thermodynamic and kinetic stability of the dopant system by mitigating *t*BP volatilization and inhibiting the aggregation/migration of Li^+^ ions. Additionally, the introduction of N3^•+^/O_2_^•−^ redox couple facilitated an efficient p‐type doping process, expediting the oxidation kinetics and yielding a substantial increase in the electrical conductivity of spiro‐OMeTAD. As a result, the devices employing this doping strategy achieved a champion PCE of 22.7% and demonstrated exceptional long‐term operational durability under both dark storage and continuous 1‐sun illumination.

To circumvent the environmental constraints associated with molecular O_2_, extensive research has focused on engineering additive oxidants that can emulate their oxidative functionality under more controlled and benign conditions. In 2022, Green et al. introduced a facile and effective doping strategy by incorporating an alkyl thiol additive, 1‐dodecanethiol (DDT), into the spiro‐OMeTAD HTL (Figure 9d). The thiol groups in DDT underwent oxidative coupling to form disulfide bonds upon exposure to external stimuli such as photoirradiation, thermal activation or alkaline stimuli, thereby functioning analogously to molecular O_2_ in facilitating the oxidation of spiro‐OMeTAD. The resultant disulfide species acted as in situ oxidants, possessing sufficient electron affinity to accept electrons from spiro‐OMeTAD molecules, driving the formation of hole polarons.^[^ ^110^ ^]^ This redox process enabled a more controllable and efficient doping pathway, significantly reducing the dependence on ambient air and its associated variability in doping kinetics and film quality. Beyond serving as an oxidizing agent, DDT imparted additional multifunctionality to HTL. Its long alkyl chain increased the hydrophobicity of the spiro‐OMeTAD layer, enhancing resistance to moisture ingress by elevating the kinetic barrier to water permeation. Moreover, DDT formed a coordination complex with LiTFSI, which restricted the ionic mobility of Li^+^ ions within the film, suppressing undesirable ion migration and dopant redistribution during device operation. These synergistic effects collectively contributed to a more stable dopant configuration and improved morphological robustness under operational stress. The resulting PSCs utilizing this DDT‐assisted doping strategy demonstrated a certified PCE of 23.1%. More importantly, the devices maintained 90% of their initial performance after 1000 h of continuous illumination under standard testing conditions.

Similarly, in 2024, Yang et al. introduced diphenyliodonium‐TFSI (IP‐TFSI), a potent organoiodonium‐based oxidant, to enable precise and air‐independent control over the oxidation state of spiro‐OMeTAD^[^ ^119^ ^]^ (Figure 9e). This approach bypassed the inherent limitations of conventional air‐mediated oxidation, such as environmental variability and sluggish kinetics. They systematically investigated the effect of various counteranions—including TFSI^−^, trifluoromethanesulfonate (OTs^−^), and hexafluorophosphate (PF_6_^−^)—on the doping dynamics of spiro‐OMeTAD. Among these, the highly delocalized TFSI^−^ anions demonstrated superior capability in stabilizing the oxidized spiro‐OMeTAD^•+^ species via effective charge delocalization and minimized coulombic interactions. Remarkably, even at a minimal dopant loading of 6 mol%, the oxidation process reached saturation within a short timeframe, and the resulting hole mobility of the IP‐TFSI‐doped spiro‐OMeTAD was ≈30‐fold greater than that of the undoped counterpart. This efficient doping strategy yielded exceptional PCEs of 25.16% and a certified efficiency of 24.85% for the PSCs, underscoring the effectiveness of the doping strategy. Furthermore, the high *T*_g_ of 120 °C imparted substantial thermal robustness to the HTL, enabling the devices to retain over 90% of their PCEs after 1000 h of thermal aging at 85 °C. Long‐term operational stability was also verified under MPPT conditions, where the devices sustained 90% of their initial PCEs for over 1370 h of continuous illumination

In 2024, Lan et al. utilized iodobenzene diacetate (ID) as an oxidizing initiator to enable rapid and controlled p‐type doping of spiro‐OMeTAD^[^ ^120^ ^]^ (Figure 9f). Leveraging its favorable redox potential (1.12 V versus Normal Hydrogen Electrode [NHE]), which is higher than that of spiro‐OMeTAD (0.89 V), ID effectively facilitated spontaneous oxidation without the need for ambient air exposure. Upon activation, ID rapidly oxidized spiro‐OMeTAD to generate the desired spiro‐OMeTAD^•+^ species under inert conditions. Simultaneously, iodobenzene, the primary byproduct, exhibited strong coordination affinity toward *t*BP, suppressing its undesired diffusion into the underlying perovskite layer—a known degradation pathway responsible for interfacial instability. This dual functionality not only enhanced doping efficiency but also contributed to the long‐term morphological and chemical stability of the device architecture. The PSCs employing this doping strategy achieved a peak PCE of 25.76% and a certified efficiency of 25.38%. Moreover, the generalizability of this oxidizing approach was validated across a range of organic HTMs beyond spiro‐OMeTAD, demonstrating its broad applicability in high‐performance optoelectronic systems.

### 4.2 Engineering Strategies to Suppress the Mobility of Ionic Dopants

**Figure 10:**
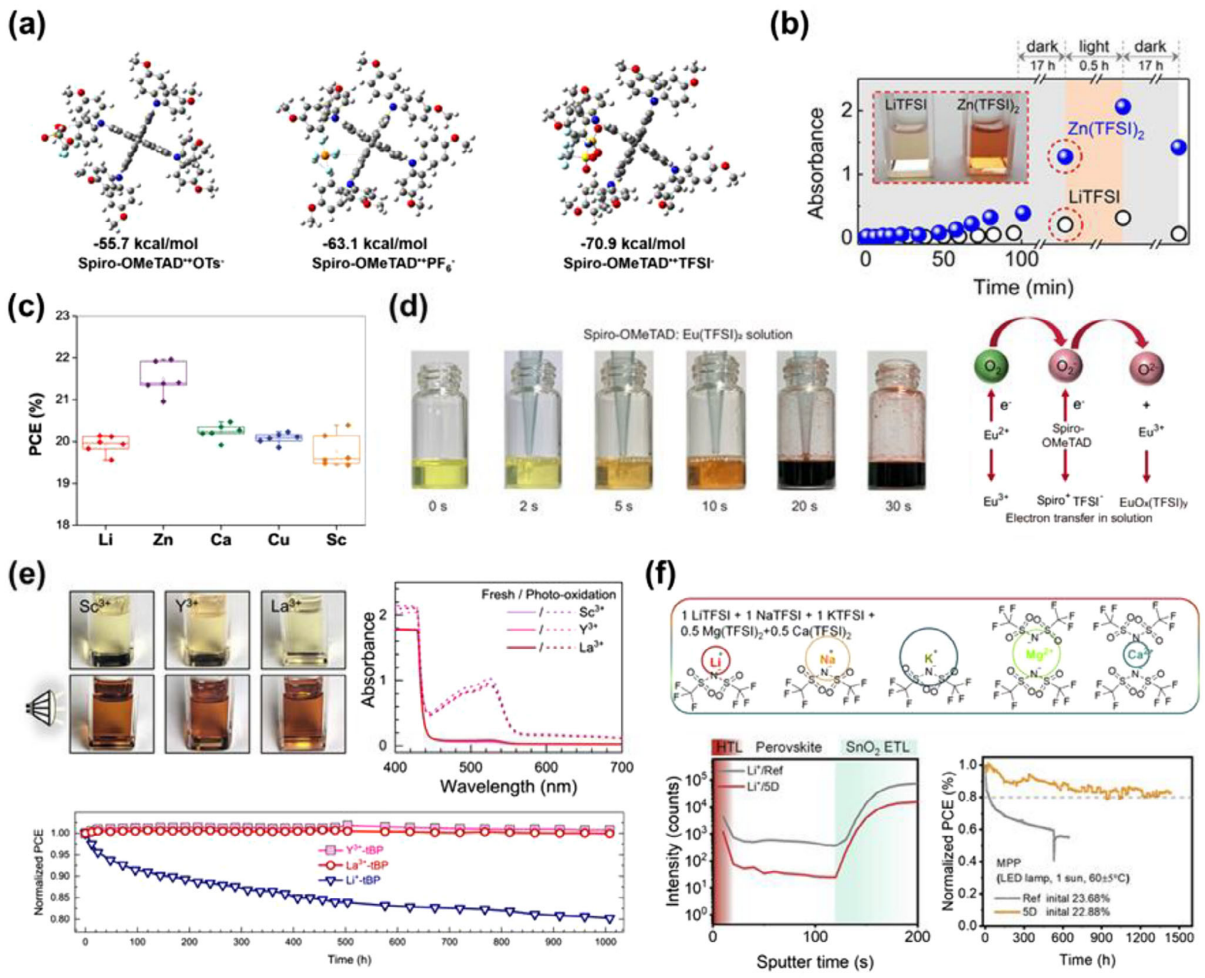
Engineering strategies to mitigate ionic dopant mobility in spiro‐OMeTAD. a) Optimized molecular geometries and corresponding binding energies of spiro‐OMeTAD^⋅+^OTs^−^, spiro‐OMeTAD^⋅+^ PF_6_^−^, and spiro‐OMeTAD^⋅+^TFSI^−^, as obtained via density functional theory (DFT) calculations. Reproduced with permission.^[^ ^119^ ^]^ Copyright 2023, Wiley‐VCH GmbH. b) Time‐dependent absorbance of oxidized spiro‐OMeTAD doped with LiTFSI (empty black symbols) and Zn(TFSI)_2_ (solid blue symbols) under an Ar atmosphere. Reproduced with permission.^[^ ^122^ ^]^ Copyright 2020, American Chemical Society. c) PCEs of devices incorporating different metal‐based dopants, M(TFSI)_n_ (M = Li, Zn, Ca, Cu, and Sc). Reproduced with permission.^[^ ^123^ ^]^ Copyright 2021, Wiley‐VCH GmbH. d) Visual color change in spiro‐OMeTAD:Eu(TFSI)_2_ solutions with increasing O_2_ bubbling duration. Schematic representation of the pre‐oxidation process and superoxide (⋅O_2_^−^)‐mediated doping mechanism induced by Eu(TFSI)_2_. Reproduced with permission.^[^ ^49^ ^]^ Copyright 2024, The Author(s). e) Photochemical oxidation behavior of spiro‐OMeTAD in the presence of different metal‐*t*BP complexes. Normalized time‐evolution of PCEs under continuous illumination over 1008 h. Reproduced with permission.^[^ ^124^ ^]^ Copyright 2024, Elsevier. f) Chemical structures of various dopants, ToF‐SIMS depth profiles of Li^+^ ion distribution across the spiro‐OMeTAD/perovskite/ETL/ITO samples, and corresponding MPPT operational stability of PSCs. Reproduced with permission.^[^ ^125^ ^]^ Copyright 2025, The Author(s).

The pursuit of enhanced doping efficiency in spiro‐OMeTAD has catalyzed the exploration of alternative p‐type doping strategies that transcend the inherent limitations associated with conventional *t*BP/LiTFSI‐based protocols. Among the dopant constituents, the TFSI^−^ anion plays a pivotal role due to its highly electronegative –CF_3_ moieties. It exerts a strong electron‐withdrawing effect, allowing extensive delocalization of the negative charge throughout the molecular backbone. This delocalization renders TFSI^−^ a non‐coordinating, weakly interacting anion that stabilizes the oxidized spiro‐OMeTAD^•+^ species without impeding hole transport (**Figure** **10**a). Owing to these attributes, the TFSI^−^ anion is predominantly employed as a dopant in most studies, thereby prompting extensive research endeavors aimed at substituting the associated cationic counterparts. Conversely, the Li^+^ cation has been increasingly scrutinized owing to its inherent drawbacks—namely, high ionic mobility and strong hygroscopicity—which collectively exacerbate ion migration under operational bias and promote moisture ingress. These effects can accelerate interfacial degradation and severely compromise the long‐term stability of PSCs. Consequently, significant research efforts have been devoted to identifying alternative cationic species that retain the beneficial doping characteristics of Li^+^ while suppressing its deleterious migration behavior.

#### 4.2.1 Metal‐TFSI^−^ Dopants

In 2018, Grätzel et al. pioneered a novel p‐type doping strategy by substituting the conventionally utilized monovalent LiTFSI with divalent Zn(TFSI)_2_ as a p‐type dopant,^[^ ^121^ ^]^ demonstrating a markedly enhanced hole mobility—exceeding that of the LiTFSI‐based system by nearly an order of magnitude. This improvement was attributed to the more effective generation and stabilization of oxidized spiro‐OMeTAD species facilitated by Zn^2+^ ions. The optimized doping environment also led to an increased built‐in electric potential and suppressed interfacial recombination—attributable to the more effective p‐type doping—culminated in a stabilized PCE of 22.0%. Notably, the devices exhibited superior operational stability under prolonged light soaking and humid conditions. Building on this progress, Hagfeldt et al. in 2020 proposed a comprehensive mechanistic framework for Zn(TFSI)_2_‐based doping, providing a detailed rationale for the deliberate selection of Zn^2+^ as the dopant cation based on its favorable electronic configuration and coordination chemistry^[^ ^122^ ^]^ (Figure 10b). Importantly, the adoption of Zn(TFSI)_2_ as a dopant circumvented degradation pathways commonly associated with hygroscopic and mobile Li^+^ ions, thereby mitigating ambiguous aging processes and extending device longevity. Inspired by these pioneering studies, subsequent research efforts have increasingly pivoted toward the exploration of alternative multivalent metal cations as dopants. In 2021, Grätzel et al. systematically investigated the doping kinetics and oxidation behavior of spiro‐OMeTAD in the presence of a variety of metal cations in M(TFSI)_n_ salts^[^ ^123^ ^]^—including Li^+^, Zn^2+^, Ca^2+^, Cu^2+^, and Sc^3+^ (Figure 10c). Through this comparative analysis, Zn(TFSI)_2_ emerged as the most promising candidate, offering a well‐balanced combination of effective p‐type doping, minimized ionic migration, and robust stability under operational stress.

Notwithstanding the substantial research endeavors aimed at developing alternative dopant systems, LiTFSI remains prevalently utilized as the benchmark dopant for high‐performance devices, owing to its proven efficacy in achieving superior PCEs. However, the intrinsic drawbacks of Li^+^ remain critical impediments to long‐term device stability. In response to these limitations, Wang et al. in 2024 introduced variant‐valence europium‐based salts, Eu(TFSI)_2_, as a promising Li‐free alternative for p‐type doping of spiro‐OMeTAD (Figure 10d). The use of Eu(TFSI)_2_ salts enabled the spontaneous formation of superoxide radicals (•O_2_^−^) through an intrinsic Eu^2+^/Eu^3+^ redox‐mediated pathway, thereby obviating the necessity for pre‐oxidation of spiro‐OMeTAD and instantly attaining high electrical conductivity.^[^ ^49^ ^]^ This redox‐driven, post‐oxidation‐independent doping strategy significantly simplified the doping procedure while simultaneously enhancing doping kinetics. The Eu(TFSI)_2_‐doped HTL delivered an exceptional PCE of 25.45% without necessitating any additional oxidation process. Furthermore, the devices demonstrated outstanding operational durability under continuous MPPT conditions, retaining 95% of their initial PCE over 1000 h of uninterrupted operation—underscoring the dual advantages of efficiency and stability.

Complementing this approach, Park et al. reported a novel photo‐induced doping method based on rare‐earth metal‐*t*BP complexes, especially Y^3+^ and La^3+^ (Figure 10e). In this strategy, efficient p‐type doping was initiated through light‐induced symmetry‐breaking charge separation, which drove the oxidation of spiro‐OMeTAD in situ.^[^ ^124^ ^]^ Crucially, this photo‐doping technique circumvented the adverse effects associated with prolonged aging, including oxidative degradation and moisture‐induced dopant migration, by enabling rapid and controlled doping under illumination. Notably, devices fabricated via this strategy exhibited negligible performance degradation under continuous 1‐sun illumination over 1000 h, further validating the efficacy of dopant design that decouples doping from ambient and temporal constraints.

In 2025, a novel doping paradigm emerged, marking a significant departure from conventional single‐metal strategies through the introduction of a synergistic co‐doping approach involving multiple metal‐based dopants. Wang et al. pioneered this mixed‐dopant architecture by incorporating a combination of metal‐TFSI salts^[^ ^125^ ^]^—including LiTFSI, KTFSI, NaTFSI, Ca(TFSI)_2_, and Mg(TFSI)_2_—into the spiro‐OMeTAD matrix (Figure 10f). This multifaceted strategy was designed to integrate complementary functionalities of each metal dopant within a single doping framework. Each individual dopant fulfills a distinct and indispensable function within the synergistic doping architecture; the Ca(TFSI)_2_ and Mg(TFSI)_2_, as divalent salts served as the primary oxidizing agents, promoting efficient p‐type doping of spiro‐OMeTAD; NaTFSI contributed to interstitial doping within the bulk perovskite lattice, improving the interface energetics and reducing interfacial recombination losses; and KTFSI while not directly involved in the oxidation of spiro‐OMeTAD, served as a catalytic agent that modulated the local chemical environment, boosting the doping efficacy of the coexisting metal‐TFSI salts. This combinatorial doping strategy yielded not only remarkable photovoltaic performance—with PCEs rivaling state‐of‐the‐art dopant systems—but also significantly conferred substantial enhancements in operational durability. The devices fabricated using this approach exhibited as a *T*_90_ operating lifetime surpassing 2000 h under thermal stress and continuous illumination conditions.

#### 4.2.2 Organic or Derivative Dopants

Beyond the conventional use of mobile small‐sized metal cations such as Li^+^ in spiro‐OMeTAD doping systems, recent studies have increasingly pivoted toward the incorporation of bulky organic cations or their functionalized derivatives as alternative cationic counterparts in dopant systems. This emerging strategy seeks to simultaneously address the challenges of ionic migration, hygroscopicity, and interfacial instability—long‐standing issues associated with alkali metal‐based dopants.

Organic cations, particularly, those bearing sterically hindered structures or delocalized charge distributions, exhibit inherently low ionic mobility and are less prone to electrochemical drift under operational bias. Their incorporation into dopant formulations mitigates detrimental ion migration phenomena that often lead to hysteresis, phase segregation, and long‐term performance degradation. Furthermore, the presence of functional moieties (e.g., quaternary ammonium, imidazolium, or pyridinium groups) in these organic dopants allows for tailored intermolecular interactions with both the spiro‐OMeTAD matrix and adjacent perovskite layers. This enables the formation of stable molecular networks, promoting more uniform dopant dispersion and enhanced morphological stability of the HTL. As the field progresses, the rational design of organically tailored cationic dopants is anticipated to play an increasingly critical role in realizing both high‐performance and long‐lifetime PSCs, especially within scalable and ambient‐compatible fabrication workflows.

**Figure 11:**
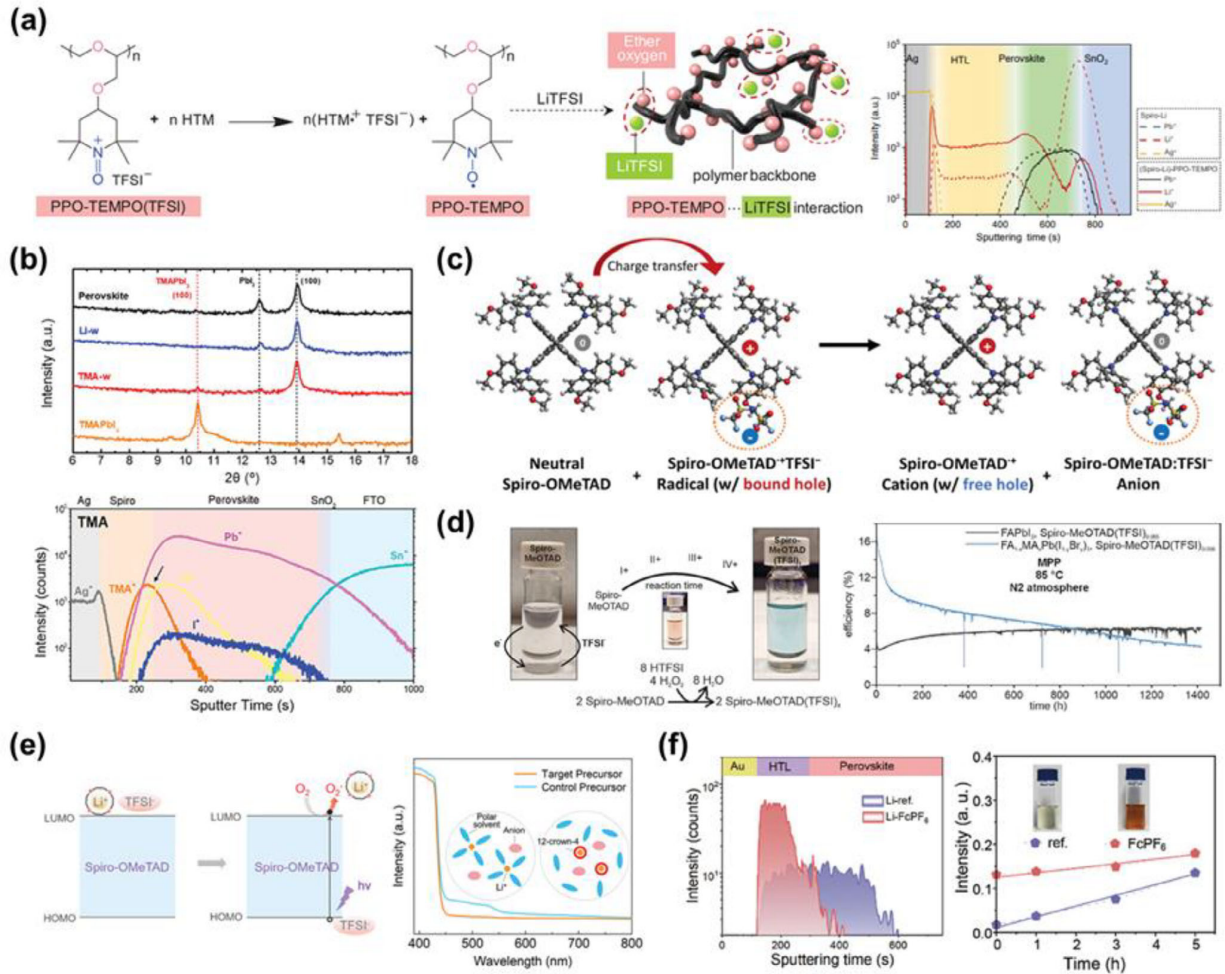
Advanced strategies for mitigating ionic dopant migration in spiro‐OMeTAD‐based HTLs. a) Schematic illustration of the doping mechanism involving PPO‐TEMPO and PPO‐TEMPO(TFSI) in organic HTMs, along with the interaction between the reduced form of PPO‐TEMPO and LiTFSI. Corresponding ToF‐SIMS depth profiles of Li^+^ ions in the SnO_2_/perovskite/spiro‐OMeTAD/Ag device structure. Reproduced with permission.^[^ ^109^ ^]^ Copyright 2023, American Association for the Advancement of Science. b) XRD patterns of the pristine perovskite film and the film following removal of the overlaid spiro‐OMeTAD:LiTFSI layer. ToF‐SIMS depth profiles of PSCs doped with TMA‐TFSI demonstrating spatial distribution of selected cationic species. Reproduced with permission.^[^ ^126^ ^]^ Copyright 2024, Wiley‐VCH GmbH. c) Illustration of the charge transfer and radical doping process mediated by spiro‐OMeTAD^⋅+^TFSI^−^ radical species. Reproduced with permission.^[^ ^115^ ^]^ Copyright 2022, American Association for the Advancement of Science. d) Oxidation mechanism of spiro‐OMeTAD with associated photographic images and a reaction schematic. MPPT operational stability of a PSC utilizing spiro‐OMeTAD(TFSI)_x_ under 85 °C continuous aging. Reproduced with permission.^[^ ^128^ ^]^ Copyright 2024, Wiley‐VCH GmbH. e) Proposed oxidation pathway of spiro‐OMeTAD in the presence of 12‐crown‐4, along with UV–vis absorption spectra of the corresponding solutions. Reproduced with permission.^[^ ^129^ ^]^ Copyright 2022, Wiley‐VCH GmbH. f) ToF‐SIMS spectra of Li^+^ ions in FcPF_6_‐PSCs after 100 h of aging at 85 °C under AM 1.5G illumination. Reproduced with permission.^[^ ^130^ ^]^ Copyright 2024, Wiley‐VCH GmbH.

In 2023, Li et al. pioneered the development of a redox‐active radical polymeric dopant featuring a polyoxyethylene backbone functionalized with (2,2,6,6‐tetramethylpiperidin‐1‐yl)oxyl moieties, termed PPO‐TEMPO^[^ ^109^ ^]^ (**Figure** **11**a). This polymeric system serves dually as a structural matrix and an intrinsic oxidizing agent for p‐type doping. Upon chemical oxidation, the neutral PPO‐TEMPO radical polymer was transformed into PPO‐TEMPO(TFSI), which was subsequently employed as an effective p‐dopant for spiro‐OMeTAD. Owing to the higher redox potential of the PPO‐TEMPO/PPO‐TEMPO(TFSI) redox couple (0.925 V versus NHE) compared to that of the spiro‐OMeTAD/spiro‐OMeTAD^•+^ pair (0.72 V vs NHE), a substantial thermodynamic driving force was provided to facilitate ultrafast hole doping. This facilitated near‐instantaneous oxidation of spiro‐OMeTAD, shortening the required doping time to just a few seconds under ambient conditions. Consequently, the resulting PSCs delivered impressive photovoltaic performance, achieving PCEs of 23.5% for 1 cm^2^ PSCs and 21.4% for 17.1 cm^2^ minimodule devices. In addition to high doping efficiency, the incorporation of PPO‐TEMPO offered considerable improvements in ionic stability. The coordination between Li^+^ ions and the TEMPO moieties effectively suppressed Li^+^ migration, mitigating common degradation pathways associated with mobile ions. This immobilization effect, contributed to outstanding operational durability, as evidenced by >3000 h of stable performance under continuous 1‐sun illumination at MPPT conditions.

To circumvent the deterioration of doping efficiency associated with the intrinsic mobility of ionic species within the device, in 2025, Jung et al. proposed a structurally simple quaternary alkylammonium salt as an alternative p‐type dopant to conventional LiTFSI. In particular, tetramethylammonium‐TFSI (TMA‐TFSI) was employed, with the TMA^+^ cations serving as the representative quaternary alkylammonium species for p‐type doping^[^ ^126^ ^]^ (Figure 11b). This alternative dopant not only served as an efficient p‐type dopant by facilitating the oxidation of spiro‐OMeTAD, achieving efficient hole doping, but also induced the formation of a 1D passivation layer via interfacial chemical interaction with the underlying perovskite film. This passivation effect contributed to suppressed non‐radiative recombination and improved interfacial energetics. Furthermore, the substitution of Li^+^ with the larger, non‐coordinating TMA^+^ cation alleviated multiple intrinsic drawbacks, including its pronounced hygroscopicity, high ionic mobility, and susceptibility to forming mobile and reactive byproducts during device operation. The deliberate integration of TMA‐TFSI‐doped spiro‐OMeTAD as the HTL imparted markedly enhanced operational stability under various external stimuli.

The implementation of radical‐based dopants obviates the necessity for conventional additives such as *t*BP and LiTFSI dopant, offering a transformative approach to overcoming the intrinsic stability limitations associated with traditional doping systems. By replacing these conventional dopants with pre‐synthesized, chemically stable radical species,^[^ ^92^, ^127^ ^]^ the doping process becomes not only more controlled and air‐independent, but also significantly more benign to the device structure and interfaces.

In 2022, Gao et al. introduced an ion‐modulated radical doping strategy designed to simultaneously optimize the electronic properties and environmental stability of spiro‐OMeTAD‐based HTLs^[^ ^115^ ^]^ (Figure 11c). This approach is centered on two key components; the first involves a pre‐synthesized, chemically stable diradical complex, spiro‐OMeTAD^2•+^(TFSI^−^)_2_, which engages in comproportionating with neutral spiro‐OMeTAD molecules to selectively generate the desired monoradical species, spiro‐OMeTAD^•+^TFSI^−^. The second component comprises 4‐*tert*‐butyl‐1‐methylpyridinium bis(trifluoromethylsulfonyl)imide (TBMP^+^TFSI^−^), an ionic salt that serves to further modulate the work function (WF) of spiro‐OMeTAD by fine‐tuning the electronic landscape of the spiro‐OMeTAD matrix. Through its incorporation, the doping level and energy‐level alignment can be finely controlled, facilitating improved charge extraction and hole transport. Importantly, this air‐independent doping methodology obviates the need for volatile *t*BP and hygroscopic LiTFSI, both of which are known to contribute to interface degradation, ion migration, and long‐term instability under moisture and heat stress. The resultant PSCs achieved a maximum PCE of 25.15%, while demonstrating exceptional operational stability under accelerated aging conditions, maintaining performance under elevated humid and heat stress conditions.

In a parallel study to the aforementioned research, Bawendi et al., in 2024, reported the rational synthesis of spiro‐OMeTAD(TFSI)_4_, a fully oxidized radical‐based dopant, which enabled the formulation of additive‐free spiro‐OMeTAD without the need for conventional metal salt additives^[^ ^128^ ^]^ (Figure 11d). The doping concentration could be judiciously modulated by blending with pristine spiro‐OMeTAD, affording tunability in both electrical conductivity and energy level alignment without compromising morphological integrity. Most notably, the implementation of this radical dopant strategy imparted a high thermal robustness, as evidenced by a *T*_g_ surpassing 115 °C, and yielded exceptional operational durability—exhibiting no discernible degradation in PCE over 1000 h of continuous MPPT under elevated thermal stress at 85 °C.

#### 4.2.3 Coordination‐Induced Sequestration of Li^+^

In 2022, Shen et al. introduced a phase‐transfer‐catalyzed LiTFSI doping strategy for spiro‐OMeTAD by employing 12‐crown‐4 ether as an efficient molecular additive^[^ ^129^ ^]^ (Figure 11e). This approach harnesses the selective host‐guest coordination interaction between Li^+^ ions and 12‐crown‐4, whose cavity size is well‐matched to the ionic radius of Li^+^. The strong binding affinity between Li^+^ ions and 12‐crown‐4 promotes the enhanced dissolution of LiTFSI in nonpolar CB by forming a crown‐Li^+^ complex that acts as a soluble phase‐transfer species. Importantly, the crowned Li^+^ ions exhibit significantly reduced ionic mobility, effectively mitigating Li^+^‐induced ion migration pathways that are detrimental to device longevity. Additionally, this complexation substantially decreases the hygroscopic nature of LiTFSI and thus increases the moisture tolerance of the doped spiro‐OMeTAD layer. The PSCs employing the 12‐crown‐4‐assisted HTL achieved an impressive PCE of 23.24%. The reduced dopant‐induced side effects—namely, suppressed ion migration and lower moisture sensitivity—conferred exceptional operational stability. The devices maintained 95.9% of their initial efficiency after 2400 h of storage under ambient conditions (30‐50% RH, 25 °C).

Recently, in 2024, Chang et al. reported a ferrocenium hexafluorophosphate (FcPF_6_)‐engineered doping strategy for spiro‐OMeTAD, aiming to circumvent the adverse effects associated with the uncontrolled migration of Li^+^ ions in PSCs^[^ ^130^ ^]^ (Figure 11f). This approach capitalizes on the strong hole‐injection capability of Fc^+^ cations, which enables the instantaneous oxidation of spiro‐OMeTAD, effectively eliminating the need for post‐oxidation treatments. Upon electron transfer, the reduced Fc species exhibit robust coordination with Li^+^, forming a novel Fc‐Li complex within the HTL. This interaction suppresses the mobility of Li^+^ ions, mitigating ion migration‐induced degradation, and structural and thermal stability of HTL. Moreover, the dissociative PF_6_^−^ anion passivates reactive iodide species on the perovskite surface, suppressing the formation of ion migration pathways and stabilizing interfacial energetics. The FcPF_6_‐integrated HTL delivered notable scalability and device performance, achieving peak PCEs of 22.13% and 20.27% in 36 cm^2^ and 100 cm^2^ perovskite solar modules (PSMs), respectively. Beyond efficiency, the FcPF_6_‐assisted Li^+^ sequestration strategy conferred exceptional operational durability, maintaining PCE retention under prolonged MPPT conditions and elevated thermal stress.

### 4.3 Rational Design Strategies for Enhancing Thermal Stability

The progressive deterioration of spiro‐OMeTAD thermal durability is predominantly attributed to the intrinsically labile nature of the oxidized spiro‐OMeTAD species and the aggravated de‐doping processes, which are primarily triggered by the presence of excessive or residual *t*BP within the spiro‐OMeTAD matrix. The volatility and chemical reactivity of *t*BP under elevated thermal conditions lead to its gradual evaporation or diffusion into adjacent layers, resulting in a destabilized doping environment. Moreover, residual *t*BP is known to chemically interact with oxidized spiro‐OMeTAD, forming electrically insulating pyridinated adducts and generating reactive *t*BP^+^ species through radical cation intermediates, which collectively accelerate dopant degradation and conductivity loss. In addition, thermal stress promotes the back‐diffusion of halide ions, particularly iodide from the perovskite layer, which further exacerbates de‐doping by reducing the oxidized state of spiro‐OMeTAD and facilitating irreversible side reactions. These synergistic degradation pathways critically undermine the thermal stability and long‐term operational reliability of HTL, especially in encapsulated device architectures exposed to elevated temperatures. Accordingly, extensive research efforts have been directed toward suppressing these degradation mechanisms by minimizing *t*BP content, designing thermally stable dopant systems, synthesizing intrinsically robust HTMs—collectively enabling stable and high‐efficiency device operation under thermal stress.

#### 4.3.1 Robust Complexation of tBP‐Li^+^

**Figure 12:**
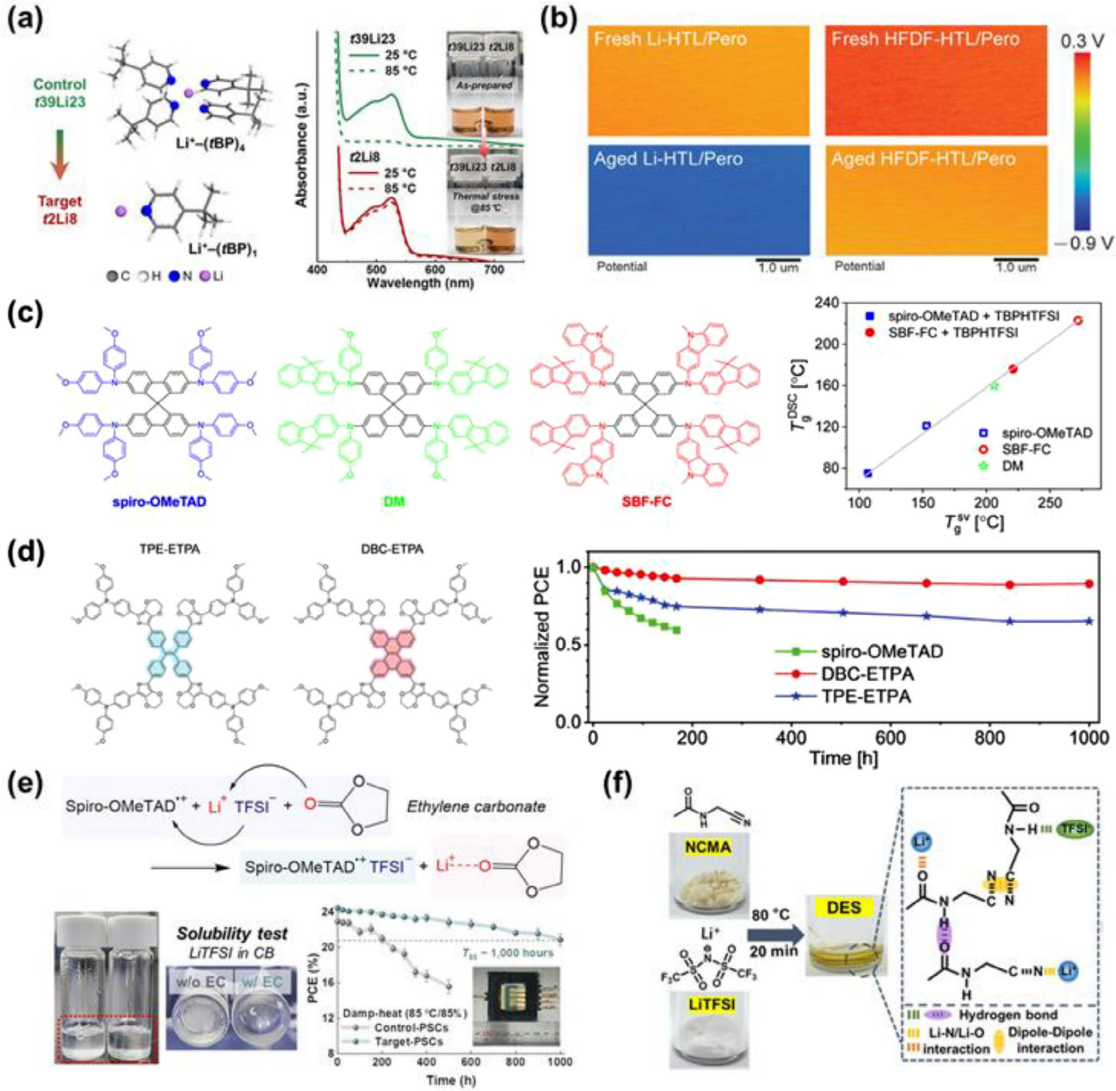
Rational material design strategies for enhancing thermal and environmental stability. a) Photographic images and corresponding UV–vis absorption spectra of spiro‐OMeTAD solutions prepared with Li^+^‐(*t*BP)_4_ and Li^+^‐(*t*BP)_1_ complexes. Reproduced with permission.^[^ ^22^ ^]^ Copyright 2025, Elsevier. b) Surface potential mapping images of HTL/perovskite acquired before (top) and after (bottom) prolonged operational aging under MPPT with AM 1.5G irradiation (100 mW cm^−2^). Reproduced with permission.^[^ ^95^ ^]^ Copyright 2022, American Association for the Advancement of Science. c) Chemical structures of spiro‐OMeTAD, DM, and SBF‐FC, along with their corresponding *T*_g_. Reproduced with permission.^[^ ^131^ ^]^ Copyright 2023, Royal Society of Chemistry. d) Molecular structures of engineered hole‐transporting semiconductors TPE‐ETPA and DBC‐ETPA. Stability of PCE over time under 85 °C thermal aging conditions. Reproduced with permission.^[^ ^132^ ^]^ Copyright 2024, Wiley‐VCH GmbH. e) Doping mechanism schematic of spiro‐OMeTAD incorporating ethylene carbonate, with corresponding damp‐heat test results (85 °C/85% RH) on encapsulated PSCs. Reproduced with permission.^[^ ^133^ ^]^ Copyright 2025, Royal Society of Chemistry. f) Schematic of the synthesis of a deep eutectic solvent (DES) from NCMA and LiTFSI, and its application as a dopant in spiro‐OMeTAD. Reproduced with permission.^[^ ^102^ ^]^ Copyright 2025, Wiley‐VCH GmbH.

Since 2011, a 6:1 molar ratio of *t*BP to LiTFSI has been ubiquitously adopted as the standard composition in spiro‐OMeTAD formulations, including in the realm of PSCs. Despite its widespread adoption, the rationale behind the universality of this ratio—and its specific relevance to perovskite‐based device architectures—has remained ambiguous and largely unexplored. In 2025, Shin et al. critically evaluated this conventional formulation and revealed its inherent limitations in achieving optimal doping efficiency^[^ ^22^ ^]^ (**Figure** **12**a). Their mechanistic investigation demonstrated that the coordination‐mediated complexation involving a single Li^+^ ion and four *t*BP molecules is characterized by weak binding interactions, which facilitate the release of unbound *t*BP. These excess *t*BP molecules not only destabilize the oxidized spiro‐OMeTAD species via de‐doping reactions but also compromise the long‐term stability of the doped HTL. Furthermore, the two uncoordinated *t*BP molecules are prone to engage in side‐reactions that exacerbate degradation under operational stress. Through a systematic optimization of the molar ratio, they proposed a revised 1:1 Li^+^‐*t*BP complexation model that exhibits significantly stronger coordination stability. This optimized formulation effectively suppresses de‐doping phenomena, thereby enhancing the persistence of the spiro‐OMeTAD^•+^ radical species and improving doping efficiency—even with substantially reduced concentrations of both *t*BP and LiTFSI. An optimally tuned dopant ratio, facilitating the formation of durable Li^+^‐*t*BP complexes, yielded a peak PCE of 26.18% and a certified PCE of 26.00%. Beyond the impressive initial performance, the elevated *T*_g_ of 105 °C imparted remarkable thermal robustness to this dopant system, with the device retaining over 85% of its initial efficiency after 1000 h of rigorous heat stress at 85 °C.

#### 4.3.2 Inhibition of Iodide Invasion

Beyond the detrimental effects of excessive or residual *t*BP within the spiro‐OMeTAD matrix, the pronounced thermal degradation driven by de‐doping phenomena is further exacerbated by the upward invasion of iodide species from the underlying perovskite layer. This iodide invasion fundamentally compromises the stability of the doped state by severing the Coulombic attraction between the oxidized spiro‐OMeTAD radical cation and its counteranion TFSI^−^, thereby precipitating a substantial degradation in hole conductivity and overall deterioration of HTL's electronic properties. To address this limitation, in 2022, Wang et al. introduced the molecular anion of 1,1,2,2,3,3‐hexafluoropropane‐1,3‐disulfonimide (HFDF^−^) as a superior alternative to TFSI^−^ (Figure 12b). Unlike TFSI^−^, HFDF^−^ exhibits stronger ionic coupling and enhanced electrostatic stabilization when paired with cationic radical species, particularly in polymer‐based HTLs.^[^ ^95^ ^]^ This strong ion pairing effectively preserves the structural and electronic integrity of the doped state, even under severe iodide exposure. Despite employing PTAA as the HTM, the incorporation HFDF^−^‐based doping chemistry conferred remarkable thermal resilience to the device architecture. The resulting n‐i‐p structured PSCs achieved a certified PCE of 23.9%, while maintaining 92% of the initial efficiency after 1000 h of continuous operation under standard 1‐sun illumination at 85 °C. This work highlights the critical importance of anion engineering in fortifying doped HTLs against halide‐induced de‐doping and enabling long‐term operational durability.

#### 4.3.3 Molecular Design for Thermally Stable HTMs

The primary origin of the thermal degradation in spiro‐OMeTAD‐based HTLs is attributed to a significant decrease in the *T*_g_ to below 70 °C, induced by the incorporation of excess dopants. This thermal softening compromises the morphological and electronic stability of HTL under operational stress. Accordingly, the development of thermally robust alternative HTMs that can maintain high‐performance photovoltaic operation requires a comprehensive strategy to overcome these intrinsic limitations. Such strategies include the molecular engineering of rigid backbones to elevate *T*_g_, the design of dopant‐free systems, and the integration of stabilizing intermolecular interactions to preserve the structural integrity of the HTL under elevated temperatures.

In 2018, Jeon et al. introduced a 9,9ʼ‐spriobifluorene‐based HTM, incorporating electron‐donating methoxylphenylfluorenamine moieties, denoted as DM, as a promising alternative to the conventional spiro‐OMeTAD HTM.^[^ ^134^ ^]^ This newly designed HTM features a finely tuned energy level alignment with the perovskite absorber, along with a significantly higher *T*_g_ of 160 °C—substantially exceeding that of spiro‐OMeTAD. These characteristics translated into enhanced photovoltaic performance, achieving a peak PCE of 23.2% in the PSCs, primarily attributed to an enhanced *V*_OC_ increase from 1.114 V to 1.144 V. Furthermore, the elevated thermal stability of DM‐based devices enabled long‐term operational reliability, with the devices retaining 95% of their initial efficiency after 500 h of thermal stress at 60 °C. In a parallel study, in 2023, Ren et al. developed a spirobifluorene‐based HTM, denoted as SBF‐FC, which incorporates a highly asymmetric fluorenylcarbazoleamine moiety as the electron‐donating unit^[^ ^131^ ^]^ (Figure 12c). This molecular design strategy enabled a favorable alignment of the HOMO energy level, comparable to that of spiro‐OMeTAD, while simultaneously achieving an elevated *T*_g_ of 176 °C. Distinct from conventional dopant systems employing *t*BP/LiTFSI, the study utilized 4‐*tert*‐butylpyridinium bis(trifluoromethanesulfonyl)imide (TBPHTFSI) as a novel organic ionic dopant, enabling effective p‐type doping without relying on hygroscopic or volatile additives. As its optimized concentration, the SBF‐FC HTM exhibited an impressive electrical conductivity of 49 µS cm^−1^, circumventing degradation pathways typically associated with the use of LiTFSI and *t*BP. The significantly elevated *T*_g_ imparted excellent thermal and morphological durability to the HTL, maintaining its electronic structure and interface integrity even under continuous thermal stress at 85 °C. As a result, PSCs employing the SBF‐FC‐based HTL yielded an average PCE of 24.5% and demonstrated exceptional thermal durability, retaining 92% of their initial efficiency after 500 h of continuous thermal stress at 85 °C.

To overcome the limitations arising from the inherently poor thermal stability of spiro‐OMeTAD, extensive efforts have been devoted to exploring alternative molecular scaffolds to replace the spirobifluorene central core unit. In 2024, Ren et al. synthesized a novel X‐shaped HTM by employing dibenzo[*g*,*p*]chrysene (DBC) as the central rigid *π*‐conjugated core and triphenylene‐ethylenedioxythiophenedimethoxytriphenylamine (ETPA) as the peripheral donor units^[^ ^132^ ^]^ (Figure 12d). The incorporation of the highly rigid DBC core significantly enhanced the molecular packing and structural stability, endowing the DBC‐ETPA‐based HTM with a remarkable elevated *T*_g_ of 202 °C. Moreover, the extended *π*‐conjugation and 3D conjugated framework facilitated efficient charge delocalization and interfacial hole transport, increasing the intrinsic hole conductivity. PSCs integrated with the DBC‐ETPA HTM exhibited a maximum PCE of 24.5%. Notably, the thermally robust molecular architecture imparted excellent long‐term operational durability, with the devices retaining 88% of their initial efficiency after 1000 h of thermal storage under 85 °C.

Similarly, in 2024, Zhang et al. developed a molecularly engineered HTM, denoted as FTPE‐ST, which incorporates a twisted conjugated dibenzo[*g*,*p*]chrysene core and coplanar 3,4‐ethylenedioxythiophene (EDOT) moieties as extended donor units.^[^ ^135^ ^]^ This molecular design strategy was devised to promote intermolecular *π–π* interactions and enable efficient multidirectional charge transport, enhancing overall hole mobility within the HTL. The FTPE‐ST‐based HTM achieved a peak PCE of 25.21% in PSCs. Furthermore, thermally resilient framework conferred outstanding operational stability, with the devices maintaining over 80% of their initial efficiency after 1000 h of continuous thermal stress at 60 °C.

#### 4.3.4 tBP‐Free Doping System

In *t*BP‐free dopant systems, a major challenge lies in the inherently poor solubility of metal‐based dopants such as LiTFSI in nonpolar solvents, which significantly impacts the morphological properties and uniformity of the resulting spiro‐OMeTAD films. While *t*BP conventionally functions as a critical morphology‐modulator additive, its volatility and chemical reactivity under thermal stress compromise long‐term thermal stability. Therefore, to achieve thermally robust PSCs, it is imperative to eliminate the use of *t*BP and to explore chemically inert or less volatile alternatives. In this context, alternative doping strategies have been extensively investigated, including the use of redox‐active radical‐based dopants and ionic liquids as efficient oxidizing agents. These *t*BP‐free approaches not only ensure the effective oxidation of spiro‐OMeTAD but also alleviate degradation pathways associated with *t*BP, enhancing both morphological integrity and thermal durability of the HTL (Table 4).

In 2025, Shin et al. introduced an innovative strategy to replace the conventional *t*BP additive by employing ethylene carbonate (EC) as an electrolyte into the spiro‐OMeTAD‐based HTL^[^ ^133^ ^]^ (Figure 12e). This approach leveraged the strong solvating capability of EC, attributable to its electronegative carbonyl functional group, which facilitates robust coordination with Li^+^ ions. Through this coordination, EC effectively enabled the dissolution of LiTFSI dopant in the nonpolar CB solvent, even in the absence of *t*BP. The improved solubility of LiTFSI not only resulted in the formation of a high density of oxidized spiro‐OMeTAD radical species but also contributed to the attainment of pinhole‐free and morphologically uniform spiro‐OMeTAD film. More importantly, the absence of *t*BP led to an enhancement of the *T*_g_, raising it to 125 °C. The formation of a thermodynamically stable EC:Li^+^ coordination complex effectively hindered the migration of ionic species under external perturbations and operational conditions. As a result, the EC‐integrated HTL not only delivered an exceptional peak PCE of 25.56% and a certified PCE of 25.51%—the highest values reported to date for *t*BP‐free HTL systems—but also demonstrated remarkable operational durability. Under harsh damp‐heat conditions (85 °C, 85% RH), the encapsulated devices retained 85% of their initial efficiency after 1000 h of continuous operation.

To improve the solubility of LiTFSI in the absence of *t*BP, in 2025, Wang et al. developed a deep eutectic solvent (DES)‐mediated doping strategy, employing a binary eutectic mixture of N‐(cyanomethyl)acetamide (NCMA) and LiTFSI^[^ ^102^ ^]^ (Figure 12f). The NCMA:LiTFSI system facilitated the dissolution of LiTFSI in nonpolar CB without the need for acetonitrile and *t*BP, primarily via multiple interaction mechanisms, including strong N─H∙∙∙O hydrogen bonding and Li─O/Li─N coordination. These interactions immobilized Li^+^ ions within the spiro‐OMeTAD matrix, effectively suppressing Li^+^ migration under operational and thermal stress conditions. Furthermore, the removal of *t*BP mitigated de‐doping pathways and raised the *T*_g_ of the HTL to 119 °C, substantially improving its thermal robustness. The optimized PSCs integrated with the DES‐based spiro‐OMeTAD HTL exhibited a maximum PCE of 25.02% and demonstrated remarkable thermal durability, maintaining over 90% of their initial PCE after 1200 h of continuous heat stress at 85 °C.

To circumvent the intrinsic limitations posed by the absence of *t*BP, research endeavors have concentrated not only on the formulation of *t*BP‐free dopants but also on the molecular engineering of non‐spiro‐OMeTAD HTMs capable of inherently obviating the requirement for extrinsic dopant‐mediated p‐type modulation. The design of non‐spiro‐architectures allows circumvention of the intrinsic limitations associated with conventional spiro‐based backbones, thereby enhancing intrinsic charge‐carrier mobility. These systems offer unparalleled structural tunability, permitting deliberate modulation of the molecular backbone, side‐chain substituents, and *π*‐conjugation pathways, which collectively facilitate precise optimization of optoelectronic properties. For the molecular engineering of non‐spiro HTMs, extensive efforts have been devoted to diversifying structural backbones beyond the canonical SBF‐ and SFX‐cores, encompassing fluorene‐,^[^ ^136^ ^]^ carbazole‐,^[^ ^137^ ^]^ triphenylamine‐,^[^ ^138^ ^]^ and cyclopentadithiophene‐^[^ ^139^ ^]^ based architectures, among others. While the photovoltaic performance of such systems has yet to rival that of doped spiro‐derivatives, their conceptual rationale is rooted in transcending the intrinsic limitations of undoped spiro‐OMeTAD analogues by imparting superior charge‐transport characteristics, elevated *T*_g_, and enhanced morphological robustness. Collectively, these molecular design strategies converge on the development of dopant‐free HTMs endowed with intrinsically fortified electrochemical and thermal resilience, thereby simultaneously mitigating fundamental constraints on the long‐term operational stability and transcending conventional *t*BP‐free approaches toward the realization of fully dopant‐independent HTM frameworks.

### 4.4 Engineering Strategies for Enhancing Humidity Resistance

The efficiency degradation of PSCs employing spiro‐OMeTAD‐based HTM is primarily attributed to the presence of volatile *t*BP and the hygroscopic nature of LiTFSI. In particular, the evaporation of *t*BP during and after film formation induces the emergence of pinholes and microvoids within the HTL, which act as vulnerable pathways for moisture ingress and severely accelerate moisture‐induced degradation processes in the perovskite layer and at the interface. In response, considerable research efforts have been directed toward suppressing the formation of such detrimental pinholes by sequestering or eliminating volatile *t*BP molecules from the spiro‐OMeTAD matrix. In parallel, the molecular engineering of HTMs with enhanced intrinsic hydrophobicity and resistance to moisture ingress has emerged as a promising strategy to mitigate humidity‐induced degradation.

#### 4.4.1 Molecular Design for Humidity‐Stable HTMs

**Figure 13:**
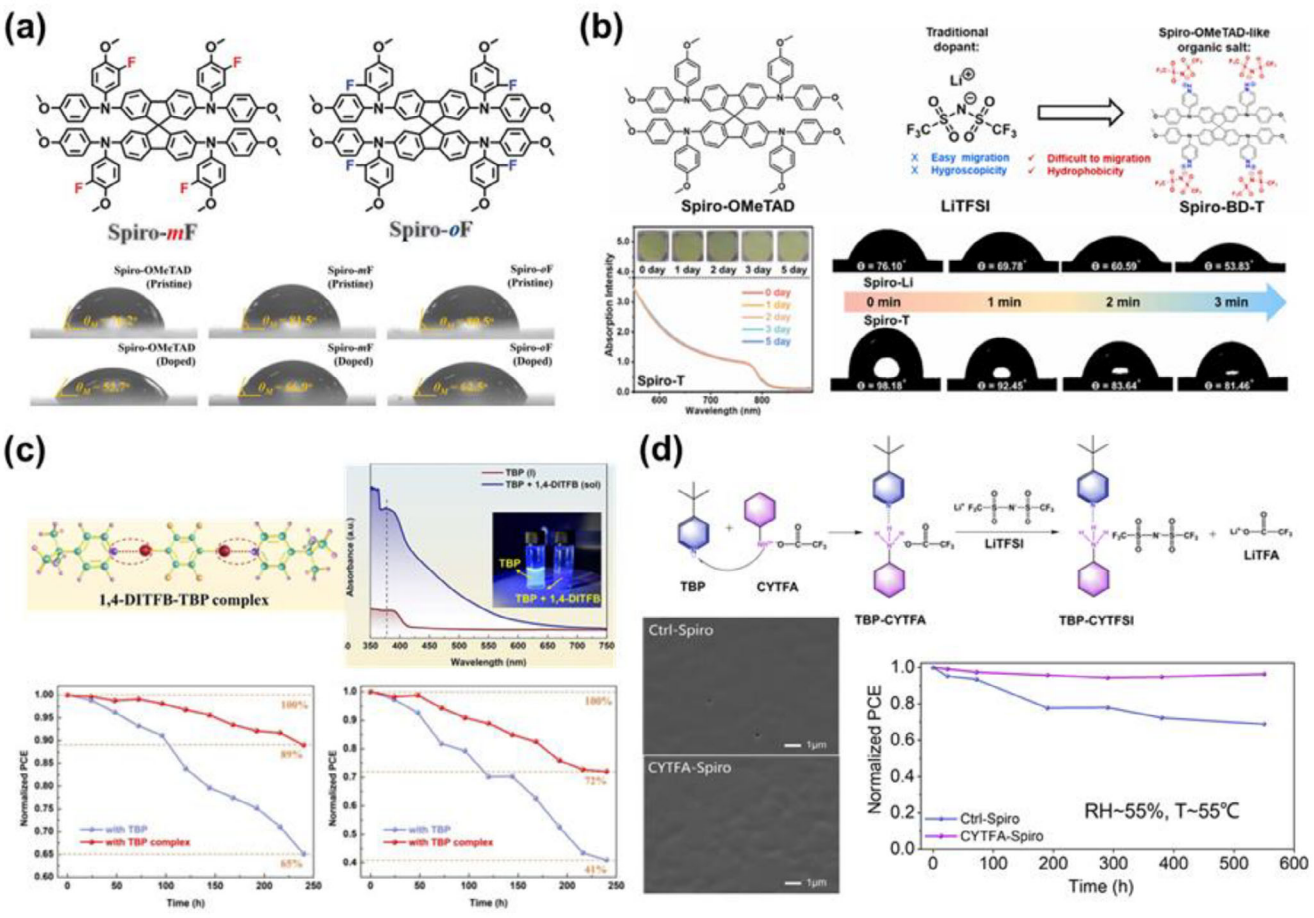
Engineering strategies to improve humidity resistance of spiro‐OMeTAD. a) Molecular structures of fluorinated spiro‐OMeTAD derivatives, Spiro‐*m*F and Spiro‐*o*F. Reproduced with permission.^[^ ^140^ ^]^ Copyright 2020, American Association for the Advancement of Science. b) Molecular structure of Spiro‐BD‐T and corresponding photographic images showing the evolution of water contact angles on perovskite surfaces coated with Spiro‐Li and Spiro‐BD‐T HTLs. Reproduced with permission.^[^ ^112^ ^]^ Copyright 2024, Elsevier. c) Schematic illustration of the interaction between TBP and 1,4‐DITFB and its effect on device stability. Time‐dependent PCE retention of TBP‐complexed devices under moderate (60 ± 5% RH) and high (85% RH) humidity conditions. Reproduced with permission.^[^ ^107^ ^]^ Copyright 2022, The Author(s). d) Mechanistic schematic illustrating the interaction of TBP and LiTFSI with CYTFA, and corresponding SEM images of the spiro‐OMeTAD‐coated perovskite films after 22 h of oxidation. Device stability under combined thermal and moisture stress (55 °C, 55% RH) with AM 1.5G illumination is also shown. Reproduced with permission.^[^ ^55^ ^]^ Copyright 2024, Royal Society of Chemistry.

In 2020, Jeong et al. synthesized a series of hydrophobic fluorinated analogs of spiro‐OMeTAD as HTMs, aiming to enhance the intrinsic moisture resistance of PSCs. This molecular design approach leverages the strong electron‐withdrawing nature of fluorine atoms, which induces permanent dipole moments along the C─F bonds. These dipoles effectively lower the HOMO energy levels, promote tighter molecular packing through enhanced intermolecular interactions, and increase the hydrophobicity of the HTM films. By systematically introducing fluorine substituents at different positions on the spiro‐OMeTAD scaffold—specifically in meta‐ (Spiro‐*m*F) and ortho‐ (Spiro‐*o*F) configurations—the authors investigated the impact of substitution position on optoelectronic properties and device performance^[^ ^140^ ^]^ (**Figure** **13**a). Among them, Spiro‐*m*F‐based PSCs achieved a remarkable PCE of 24.82% and a certified PCE of 24.64%, with only 0.3 V voltage loss. More importantly, the fluorinated HTM films demonstrated substantial resistance to moisture ingress without encapsulation. Upon exposure to high RH conditions, they demonstrated the long‐term stability without encapsulation on exposure to high relative humidity (50% RH) conditions, Spiro‐*m*F‐based devices retained 87% of their initial efficiency after 500 h.

Yang et al., in 2023, developed a Spiro‐OMeTAD‐like organic salt, termed Spiro‐BD‐T, which incorporates a large‐sized cation consisting of a spirobifluorene core tethered with phenylpyridin‐4‐amine (BD), and paired with the TFSI^−^ anion.^[^ ^112^ ^]^ This rational molecular design was intended to eliminate the need for hygroscopic Li^+^, which is known to exacerbate degradation phenomena in PSCs, particularly under humid environments. By circumventing the use of Li^+^, the detrimental effects associated with its ionic migration were effectively mitigated. Furthermore, the TFSI^−^ anion in Spiro‐BD‐T facilitated the spontaneous oxidation of spiro‐OMeTAD, thereby enabling efficient hole transport without the reliance on external dopants (Figure 13b). Devices incorporating Spiro‐BD‐T achieved a maximum PCE of 24.2% and exhibited remarkable operational durability. Notably, the bulky hydrophobic cation also played a crucial role in suppressing moisture ingress through micro‐sized pinholes formed by the gradual evaporation of *t*BP, significantly enhancing the humidity stability of the devices. As a result, the devices maintained 93% of their initial PCE after 1000 h of aging under humid conditions.

#### 4.4.2 Attenuation of the Volatile Nature of tBP

In 2022, Ren et al. elucidated the multifaceted detrimental effects of *t*BP, highlighting not only its adverse influence on the underlying perovskite layer but also its tendency to destabilize the oxidized state of spiro‐OMeTAD by promoting undesirable de‐doping dynamics. In response to these issues, they pioneered a halogen‐bonding‐mediated strategy to suppress *t*BP volatilization through the introduction of 1,4‐diiodotetrafluorobenzene (1,4‐DITFB) (Figure 13c). The strong electron‐withdrawing nature of fluorine atoms significantly enhances the electrophilicity of the iodine centers in 1,4‐DITFB molecule, thereby rendering it an efficient halogen bond donor capable of forming a stable 1,4‐DITFB‐*t*BP complex.^[^ ^107^ ^]^ This complexation effectively mitigates *t*BP volatility and its associated detrimental effects on the device performance. Accordingly, it not only minimizes the deleterious degradation of the underlying perovskite layer but also facilitates the formation of a uniformly distributed spiro‐OMeTAD film devoid of microscale pinholes. Beyond halogen and coordination bonding strategies, hydrogen bonding was also deliberately exploited as a molecular design principle to identify optimal additive candidates capable of further stabilizing *t*BP within the spiro‐OMeTAD framework. Such multivalent interaction engineering significantly enhanced the thermal and environmental stability of the HTM. As a result of these synergistic molecular stabilization strategies, the resultant PSCs achieved a remarkable PCE of 23.03% and demonstrated excellent operational durability under humid conditions. Specifically, the devices retained 89% and 72% of their initial efficiency after 240 h of operation at 60 ± 5% RH and 85% RH, respectively.

In 2024, Pan et al. pioneered the integration of an organic molten salt dopant, cyclohexylamine trifluoroacetic acid (CYTFA), to enhance the long‐term chemical and morphological stability of doped spiro‐OMeTAD^[^ ^55^ ^]^ (Figure 13d). The incorporation of CYTFA into the spiro‐OMeTAD matrix induces robust intermolecular interactions, primarily through hydrogen bonding and ionic coordination. Specifically, the amine moiety of CY^+^ participates in N─H∙∙∙N hydrogen bonding with *t*BP, while the TFA^−^ can interact electrostatically with Li^+^ ions. As a result of these multivalent interactions, the CYTFA‐based spiro‐OMeTAD layer exhibited a flat and compact morphology with significantly reduced surface roughness and the complete elimination of microscale pinholes, thereby blocking penetration pathways for moisture. Moreover, CYTFA doping was found to substantially enhance the hole mobility of spiro‐OMeTAD by nearly an order of magnitude. These synergistic effects collectively contributed to the realization of high‐performance PSCs, which achieved an exceptional peak PCE of 25.80% for the PSCs. In addition to their outstanding efficiency, the CYTFA‐based PSCs demonstrated remarkable hygrothermal stability under accelerated aging conditions of 55 ± 2 °C and 55 ± 5% RH conditions, maintaining 95% of their initial PCE after 550 h of continuous operation.

### 4.5 Emerging Dopant‐Free and Cost‐Effective HTMs

The synthesis and development of novel HTMs are driven not only by the imperative to establish dopant‐free HTM systems but also by the pressing need to mitigate the prohibitively high synthesis cost and intricate multi‐step fabrication associated with conventional spiro‐OMeTAD—factors that severely hinder its practical deployment and large‐scale manufacturability in PSCs. These constraints inflate overall production expenses and impose scalability limitations, thereby intensifying the search for alternative HTMs featuring streamlined synthetic pathways, reduced material costs, and optoelectronic properties comparable to or surpassing those of spiro‐OMeTAD.

The market price of spiro‐OMeTAD typically ranges from ≈60 to 125 $ g^−1^, contingent upon material purity and procurement scale, while research‐grade or ultra‐high‐purity (>99.5%) batches can command prices as high as 500–700 $ g^−1^. This substantial cost premium stems from its intrinsically multi‐step and low‐yield synthesis, compounded by the use of expensive specialty precursors and rigorous purification protocols required to meet the performance standards demanded in high‐efficiency PSCs.

In pursuit of next‐generation HTMs that can combine low‐cost fabrication with high PCE, substantial research efforts have been devoted to molecular design, synthetic innovation, and process optimization of alternative materials (**Table** **5**). In 2024, Zhou et al. reported T2, a multifunctional HTM incorporating an SFX core, offering a more favorable energy level alignment for hole extraction than spiro‐OMeTAD and achieving a PCE of 26.41%, thereby surpassing the benchmark performance. Notably, its synthesis can be accomplished at a cost of merely 14 $ g^−1^, representing a dramatic reduction compared to spiro‐OMeTAD and effectively addressing a major economic barrier to large‐scale PSC production. Most recently, in 2025, Urieta‐Mora et al. developed PTZ‐Fl, a cost‐effective spiro‐phenothiazine‐based HTMs that achieved a PCE of 25.8% while demonstrating scalability through compatibility with large‐area fabrication methodologies.

**Table 5:** Performance metrics and cost comparison of recently reported dopant‐free, cost‐effective HTMs in PSCs.

Published year	HTMs	Cost	Photovoltaic parameters		Refs.
			PCE [%]	Scalability	
2017.12	TPE (FASnI_3_)	30 $ g^−1^	7.23	–	[193]
2019.11	BDT‐4D (FASnI_3_)	18.6 $ g^−1^	7.59	–	[194]
2022.05	DImBT‐4D	15.7 $ g^−1^	20.11	–	[195]
2022.09	MDA4	93.4 $ g^−1^	22.67	–	[196]
2023.04	mCl‐SFXDA	10.9 $ g^−1^	22.14	–	[197]
2024.01	SFX‐3	32 $ g^−1^	22.42	–	[198]
2024.02	SP‐SMe	26.254 $ g^−1^	21.95	–	[174]
2024.06	T2	14 $ g^−1^	26.41	21.45 (14.4 cm^2^)	[176]
2024.11	mDPA‐SFX	13.4 $ g^−1^	24.8	19.26 (12.95 cm^2^)	[177]
2025.05	TzTzTPA‐NH	23.09 $ g^−1^	24.2	–	[199]
2025.06	PTZ‐Fl	56.94 $ g^−1^ (49 € g^−1^)	25.75	22.07 (25 cm^2^)	[200]

From a scalability standpoint, the enhanced solubility characteristics and pronounced ambient stability of these next‐generation spiro‐backbone HTMs underpin their compatibility for large‐area, solution‐processed deposition techniques—such as slot‐die and blade coating—that are indispensable for industrial‐scale module fabrication. The convergence of simplified synthesis, optimized cost‐to‐performance metrics, and robust process compatibility represents a pivotal breakthrough in overcoming the enduring challenges of spiro‐OMeTAD's synthetic complexity and elevated cost, thereby advancing high‐efficiency perovskite photovoltaics toward commercial realization at the gigawatt scale.

## 5 Conclusion and Perspective

Spiro‐OMeTAD‐based HTLs have been instrumental in propelling the PCEs of n‐i‐p structured PSCs to unprecedented levels, establishing themselves as the benchmark HTL in the domain of high‐performance photovoltaic architectures. The advantageous alignment between the HOMO of spiro‐OMeTAD and the valence band of perovskite absorbers, coupled with its compatibility with oxidative p‐type doping, has enabled efficient hole extraction and minimized interfacial recombination. However, the widespread reliance on a conventional dopant system—comprising LiTFSI and *t*BP—has surfaced as a critical bottleneck, severely compromising the long‐term operational stability, environmental robustness, and scalability of PSCs.

This review has elucidated the multifaceted degradation pathways originating from the dopant constituents. The hygroscopicity of LiTFSI facilitates moisture ingress, leading to hydrolysis, ion migration, and interfacial decomposition. Meanwhile, the volatility and chemical reactivity of *t*BP induce morphological instability, de‐doping of the oxidized spiro‐OMeTAD species, and exacerbated degradation of the underlying perovskite layer. Moreover, the slow and oxygen‐dependent post‐oxidation doping process necessitates prolonged ambient aging, rendering the doping kinetics susceptible to uncontrollable environmental variables and introducing irreproducibility across fabrication batches.

In light of these critical limitations, extensive research efforts have been devoted to advancing the compositional, structural, and interfacial engineering of the spiro‐OMeTAD HTL system. Notable progress spans several complementary directions. 1) Post‐oxidation‐independent doping strategies—utilizing pre‐synthesized radical salts, organoiodonium‐based oxidants, and redox‐active molten salts—have enabled air‐free, rapid, and uniform p‐doping of spiro‐OMeTAD. These approaches not only eliminate environmental variability but also accelerate doping kinetics while circumventing *t*BP‐ and O_2_‐induced instability. 2) Cationic substitution of Li^+^ with multivalent metal ions (e.g., Zn^2+^, Eu^2+^, Sc^3+^) or bulky organic cations (e.g., tetramethylammonium, pyridinium derivatives) has proven effective in mitigating ionic mobility, enhancing interfacial passivation, and suppressing dopant diffusion‐induced degradation. 3) Furthermore, intermolecular dopant stabilization via halogen bonding, hydrogen bonding, and coordination complexation has provided robust molecular‐level retention of *t*BP and Li^+^ within the spiro‐OMeTAD matrix, facilitating the formation of compact, defect‐suppressed HTL films devoid of microscale pinholes—thereby impeding the permeation of moisture and oxygen.

4) In parallel, molecular design of thermally resilient HTMs—incorporating high‐*T*_g_ backbones and dopant‐free or minimally doped frameworks—has yielded HTLs with superior morphological integrity and suppressed degradation under elevated thermal stress, with some systems maintaining over 90% of their initial PCE after 1000 h at 85 °C.^[^ ^201^ ^]^ 5) *t*BP‐free doping systems, employing alternative dopant solvents (e.g., ethylene carbonate, deep eutectic solvents), as well as radical‐mediated doping strategies, have successfully decoupled the film morphology control from volatile species, enabling stable device operation without compromising conductivity or energy‐level alignment. From a molecular design standpoint, emerging research trajectories encompass the development of HTMs predicated not only on SBF‐ and SFX‐based cores but also on architectures featuring linear backbones^[^ ^171^ ^]^ and spiro‐fluorene/heterocyclic core^[^ ^202^ ^]^ motifs, thereby delineating novel paradigms for enhanced device performance and operational stability.

Additional advances include 6) molecular fluorination of spiro‐OMeTAD derivatives, which imparts hydrophobicity and reinforces environmental resistance under humid conditions while retaining high photovoltaic performance.^[^ ^203^ ^]^ In the context of the growing prominence of organic self‐assembled monolayers (SAMs) as HTMs in inverted‐structured PSCs, molecular design strategies targeting dopant‐free HTMs with enhanced thermal and moisture stability—exemplified by spiro‐acid SAMs^[^ ^204^ ^]^—offer valuable guidance for future directions and potential avenues for expansion in inverted PSCs.

Importantly, these strategies go well beyond merely suppressing degradation. Rather, they open new pathways to boost device performance through improved film morphology, more homogeneous dopant distribution, and suppressed interfacial recombination. Thus, the dichotomy between performance and stability, long perceived as a trade‐off, is gradually being dismantled. Looking ahead, the continued refinement of the spiro‐OMeTAD HTL system should aim to transcend the limitations of legacy dopant formulations by embracing interdisciplinary design principles—integrating insights from solid‐state chemistry, materials science, and device engineering. As perovskite photovoltaics progress toward commercialization, achieving scalable, stable, and reproducible HTL systems will be paramount.

In this context, organic HTMs—by virtue of their intrinsically tunable optoelectronic properties, facile solution processability, and inherent compatibility with flexible, stretchable, and semi‐transparent device architectures—are exceptionally well‐positioned for integration into emerging photovoltaic application domains. Critically, their ability to enable conformal coating on diverse substrates while maintaining mechanical integrity under bending or thermal cycling renders them particularly advantageous for building‐integrated photovoltaics (BIPV), vehicle‐integrated photovoltaics (VIPV), and portable or wearable energy‐harvesting devices. In such application domains, lightweight form factors, design versatility, and aesthetic adaptability are paramount, and the use of organic HTMs can enable the realization of high‐performance, visually compelling, and architecturally harmonious energy systems without compromising device stability or efficiency.

In recent years, spiro‐OMeTAD has maintained its position as the most widely adopted HTM for high‐efficiency PSCs, owing to its exceptional hole‐extraction kinetics and favorable energetic alignment with the perovskite absorber. A broad repertoire of design strategies—spanning advanced dopant engineering, heteroatomic substitution, molecular framework modulation, and interfacial passivation—has propelled n‐i‐p architectures to record‐setting PCEs. Yet, these advances have been realized predominantly at the laboratory scale under small‐area, single‐cell configurations, thereby exposing a pronounced gap between record performance and industrial translatability. Performance reproducibility, environmental and operational durability, and manufacturing compatibility remain conspicuously fragile when extended to large‐area module fabrication and protracted field operation. Furthermore, the stability gains frequently reported in the literature have been corroborated largely under accelerated aging regimens or tightly constrained environmental parameters, rendering extrapolation to authentic deployment conditions—where extrinsic stressors and process variability are inescapable—both nontrivial and potentially over‐optimistic.

From a techno‐economic standpoint, the path toward commercialization demands HTMs that simultaneously attenuate efficiency losses and substantially lower raw material and processing costs. However, most state‐of‐the‐art spiro‐OMeTAD derivatives remain tethered to synthetically elaborate, cost‐intensive chemistries. The transition to industrially relevant scales introduces further systemic bottlenecks, including the maintenance of deposition uniformly, nanometric precision in film‐thickness control, and spatial homogeneity of dopant incorporation—while the establishment of rigorous, standardized manufacturing protocols and industry‐aligned design frameworks remains at an early stage.

Consequently, the continued relevance of spiro‐OMeTAD in next‐generation PSC architectures will depend less on its past achievements and more on its ability to adapt to emerging materials and device‐level challenges, ultimately overcoming the long‐standing trade‐offs between efficiency, stability, and manufacturability. Strategic convergence of compositional engineering, unconventional doping paradigms, and interdisciplinary design principles—integrating advances in solid‐state chemistry, thin‐film processing science, and photovoltaic device physics—may enable spiro‐OMeTAD and its tailored derivatives to persist as cornerstone enablers in the development of scalable, operationally stable, and commercially tenable perovskite photovoltaic technologies.

## Conflict of Interest

The authors declare no conflict of interest.
